# Estrogenic activity of mixtures in the Salish Sea: The use of high throughput toxicity data with chemical information from fish bile and other matrices

**DOI:** 10.1371/journal.pone.0323865

**Published:** 2025-07-09

**Authors:** Maya Faber, C. Andrew James, Louisa B. Harding, Denis A. M. da Silva, Ruth M. Sofield

**Affiliations:** 1 Environmental Sciences Department, Western Washington University, Bellingham, Washington, United States of America; 2 Center for Urban Waters, University of Washington Tacoma, Washington, United States of America; 3 Washington Department of Fish and Wildlife, Olympia, Washington, United States of America; 4 Environmental and Fisheries Sciences Division, Northwest Fisheries Science Center, National Marine Fisheries Service, National Oceanic and Atmospheric Administration, Seattle, Washington, United States of America; University of Tehran, IRAN, ISLAMIC REPUBLIC OF

## Abstract

A subset of anthropogenic chemicals known as contaminants of emerging concern (CECs), are released into aquatic environments through human activities. CECs occur in mixtures, and some may share a common mode of action such as estrogen receptor agonism, which lead to reproductive disturbances in fish. In this study, the estrogenic activity of mixtures was assessed with *in vitro* high throughput data, which expanded the number of chemicals included in the evaluation. Data were compiled from 16 studies, analyzing 387 CECs (19 estrogen agonists detected), across various matrices including water, wastewater treatment plant effluent, fish and mussel tissue, and fish bile. Novel estrogenic mixture thresholds in water and bile were developed. In one application of the bile thresholds, field sites with elevated exogenous estrogenic activity were identified; thresholds were qualitatively validated using field measures of organism response. Using validated water and bile thresholds in a second application, samples were evaluated to identify mixtures with high, medium, and low estrogenic activity, and individual chemicals were prioritized from those mixtures. Prioritized chemicals were identified as drivers of estrogenic activity (individually exceeding the threshold) or as major or minor contributors (resulting in an exceedance only when combined with other chemicals). Among fish bile samples with medium or high estrogenic activity, 62% of mixture response was explained by chemical drivers rather than mixtures of contributing chemicals. The primary drivers were: estrone, 17β-estradiol, and to some extent, estriol. Bisphenol A was identified as a major contributor.

## Introduction

Anthropogenic activities related to agriculture, urbanization, and industrialization introduce a diverse array of chemicals into the aquatic environment. Contaminants of emerging concern (CECs) constitute a subset of anthropogenic chemicals that are inadequately understood in terms of their environmental occurrence and toxicological impact and typically lack regulation [1]. CECs are increasingly recognized for their potential to cause adverse effects in aquatic organisms. One category of CECs, endocrine disrupting chemicals (EDCs), has been frequently investigated because the endocrine system is conserved across most vertebrate species [2], and disruption can impact development [3,4], reproduction [5–7], and behavior [8].

Estrogenic endocrine disrupting chemicals (e-EDCs) are a subclass of EDCs that are of concern because of their ability to mimic endogenous estrogens and interact with the estrogen receptor, thereby disrupting estrogen receptor signaling [9]. Exogenous estrogens present in the environment, both natural (e.g., 17β-estradiol (E2), estrone (E1), estriol (E3)) and synthetic (e.g., 17α-ethynylestradiol (EE2)), are considered e-EDCs, as are anthropogenic chemicals such as industrial phenolic compounds (e.g., bisphenol A (BPA), octylphenol, and 4-nonylphenol) [10]. There is substantial evidence of impacts from e-EDC exposures [7,9,11,12]. Laboratory and field exposure to e-EDCs at environmentally relevant concentrations have been associated with vitellogenin (Vtg) induction (a common biomarker of exposure) in male fish and juveniles [7,13,14]. e-EDC exposures have also been associated with reproductive impairments, including compromised gamete and sperm quality, disrupted reproductive timing, reduced reproductive success, and gonadal intersex [7,12,15,16].

In the environment, chemicals occur as complex mixtures. Traditional toxicological methods employed to establish water quality benchmarks focus on single-chemical evaluations. However, effects can be higher when co-occurring chemicals are considered, especially those with shared modes of action [6,17–19]. Therefore, even when individual chemical concentrations are below effects thresholds, the combined effects of chemicals in mixtures may result in a biological response [20,21].

Assessing the effects of chemical mixtures in the environment is challenging because of the interactions between co-occurring contaminants and the constantly changing mixture composition [17,19,21]. New Approach Methodologies (NAMs), such as *in vitro* effect-based methods, estimate total estrogenic activity of an environmental sample, but do not differentiate between individual contributing chemicals. Another common approach for estimating mixture effects is the 17β-estradiol equivalency quotient (EEQ) [7,9,22] which predicts estrogenic mixture activity by summing the normalized concentrations of individual e-EDCs using estradiol equivalency factors (EEFs) derived from assays like the Yeast Estrogen Screen [7,9]. However, EEFs have only been developed for a small number of chemicals, including steroidal estrogens and some phenolic chemicals that can mimic estrogens [7,9], which is limiting, considering the substantial number and diverse types of CECs that are potentially estrogenic [23]. Emerging NAMs, such as *in vitro* high-throughput screening (HTS) assays, use automated technology to screen large numbers of chemicals for a specific biological activity [24]. *In vitro* HTS information can fill data gaps such as the limited availability of EEFs [25], and can be used to assess chemicals with shared modes of action which is particularly valuable for mixture evaluation.

Large-scale *in vitro* HTS programs known as Tox21 [26,27] and ToxCast [28], hereafter referred to collectively as ToxCast, capture information from *in vitro* HTS bioassays. ToxCast includes *in vitro* HTS data for over 9,000 chemicals for more than 300 unique signaling pathways, including data for several chemicals lacking traditional health or environmental effects data [29]. In ToxCast assays, primarily mammalian cells or isolated proteins are exposed to chemicals, and changes in biological activity, such as receptor agonism or antagonism, or generalized disruption leading to cytotoxicity [30], are measured [2,18]. The biological target identified as the most well-represented in ToxCast is the estrogen receptor (ER), with extensive assay coverage [29]. Additionally, within the suite of chemicals tested in ToxCast, e-EDCs are common. Recent studies have provided evidence that the use of ER agonist assays in ToxCast can be effective for identifying e-EDCs [23,30–32]. For example, Judson et al. [23] created a model using ToxCast data to predict ER agonist activity for over a thousand chemicals, which correctly identified ER agonists known to perturb the ER pathway (e.g., receptor binding, receptor dimerization, DNA binding, RNA transcription, protein production, and ER-induced proliferation) suggesting the model may be used to identify new e-EDCs. While ToxCast assays are primarily mammalian-based, Dreier et al. [31] found that ToxCast ER agonist assays were useful in predicting reproductive outcomes in fish, specifically Vtg induction.

Exposure-activity ratios (EARs) offer another application of ToxCast data. EARs are ratios of chemical concentrations in the environment and the chemical-specific response measured in the ToxCast assays [29,33]. The preferred assay response measure is the activity concentration at cutoff (ACC) [18,29,34,35], which is the minimum concentration of an individual chemical that can produce a measurable response in an *in vitro* assay [29,36–38]. A chemical is of concern if the EAR exceeds an established threshold, suggesting the potential for biological activity *in vivo* [34]. EARs are a valuable tool for risk-based evaluations of individual chemicals, enabling efficient and cost effective screening and prioritization of chemicals [1,34,39–41]. Additionally, EARs can be used in assessing chemical mixtures with a focus on all modes of action or specific modes of action depending on which assay results are used in the calculation of EARs [18,34,35,42].

In previous research applying EARs for chemical screening and prioritization, data was typically from a single environmental matrix, predominantly water. One exception is work conducted in Puget Sound, WA, the second largest estuary in the United States. James et al. [1] compiled chemical occurrence data from multiple environmental matrices, including marine water, wastewater treatment plant (WWTP) effluent, and fish and mussel tissues to screen and prioritize individual CECs. The use of multiple matrices provides a more complete understanding of chemical occurrence and concentration. This is in part because some chemicals evaluated in this work were only analyzed in certain matrices. Also, in general, chemicals will distribute differently across matrices based on their chemical and physical properties and, in the case of biota, based on what species was analyzed as different species have different toxicokinetics, geographic distributions, and life histories. Although not considered in the previous work [1], various tissues within species accumulate chemicals differently. For example, there is preferential accumulation of e-EDCs in fish bile because they are often eliminated through biliary excretion [43–46]. This physiological process led to higher concentrations of e-EDCs observed in fish bile compared to other tissues such as plasma, muscle, brain, skin, and gill [47,48]. Despite the recognition of the importance of various sampling matrices, thresholds for estrogenic chemical mixtures in different matrices remain undeveloped.

The aim of the present study was to develop, apply, and validate a methodology for assessing the estrogenic potential of e-EDCs in mixtures in the marine environment, utilizing ToxCast data. Estrogenic EAR (e-EAR) thresholds were developed in water and bile relative to *in vitro* HTS data focused solely on estrogenic activity. In the first application of the e-EAR thresholds for bile, sites were identified where elevated exogenous estrogenic exposures may be occurring. Since this is the first effort to identify fish bile thresholds, a qualitative validation was conducted using published field measures of organism response related to exogenous estrogen exposures (*in vivo*), including Vtg induction in male fish and reproductive maturity of females. In a separate application, environmental samples were compared to water or bile-based thresholds to classify samples based on the mixture’s estrogenic potential. Individual chemicals from those mixtures were then prioritized for additional monitoring and study. This work complements the previous James et al. [1] individual chemical prioritization by considering estrogenic responses in mixtures and including additional biological samples from fish bile.

### Experimental methods

#### Environmental monitoring data.

Regional chemical monitoring data from the Salish Sea were compiled from 16 individual studies from local, state, and federal sources (S1 Table). The Salish Sea is an inland sea that includes the marine waterways and watersheds of both British Columbia, Canada and Washington, United States [49]. This study focused on the Washington Salish Sea, which encompasses the Strait of Juan de Fuca, Puget Sound, and a portion of the Strait of Georgia. Data were from multiple environmental matrices, including water, WWTP effluent, mussel tissue (whole body), fish tissue (whole body/filet), and fish bile collected from 2008 to 2021. For clarity, whole body and filet tissues are hereafter referred to as “tissue”; when bile is the tissue of interest, it is referred to as “bile”. The use of multiple studies maximized the amount of data available for analysis and geographic coverage of contaminant occurrence data. As sampling was not coordinated under a single sampling program, the study designs, analytical methods, and suite of analyzed chemicals varied and were not always focused on e-EDCs. Methods from James et al. [1] were followed for quality assurance and blank correction for all data (S1 Text).

The compiled dataset included more than 1,000 samples and 387 unique compounds, such as hormones (n = 29), per- and polyfluoroalkyl substances (PFAS; n = 40), phthalates (n = 7), bisphenols (n = 5), antibiotics (n = 53), flame retardants (n = 30), and pharmaceuticals and personal care products (n = 86), with different compounds measured depending on the sample and matrix (S2 Table). Marine water samples (n = 134) were collected from estuarine, nearshore, and pelagic environments. WWTP effluent samples (n = 9) were collected from five facilities in Puget Sound. Tissue samples were collected from bay mussels (*Mytilus trossulus*; n = 75) and multiple species of fish, including Smallmouth bass (*Micropterus dolomieu;* n = 9), Pacific staghorn sculpin (*Leptocottus armatus*; n = 5), Pacific herring (*Clupea pallasii*; n = 20), subadult resident and juvenile Chinook salmon (*Oncorhynchus tshawytscha*; n = 74 and 34, respectively), English sole (*Parophrys vetulus*; n = 123*),* Pacific sand lance (*Ammodytes personatus*; n = 10), Quillback rockfish (*Sebastes maliger*; n = 1), and Brown rockfish (*Sebastes auriculatus*; n = 18). Bile samples were collected from English sole (n = 500). Samples were marine or estuarine except the Smallmouth bass collected from an estuary-adjacent freshwater lake and select juvenile Chinook salmon samples collected from five upstream freshwater sites. Information on permits obtained for this work can be found in S1 Table.

**Fig 1:**
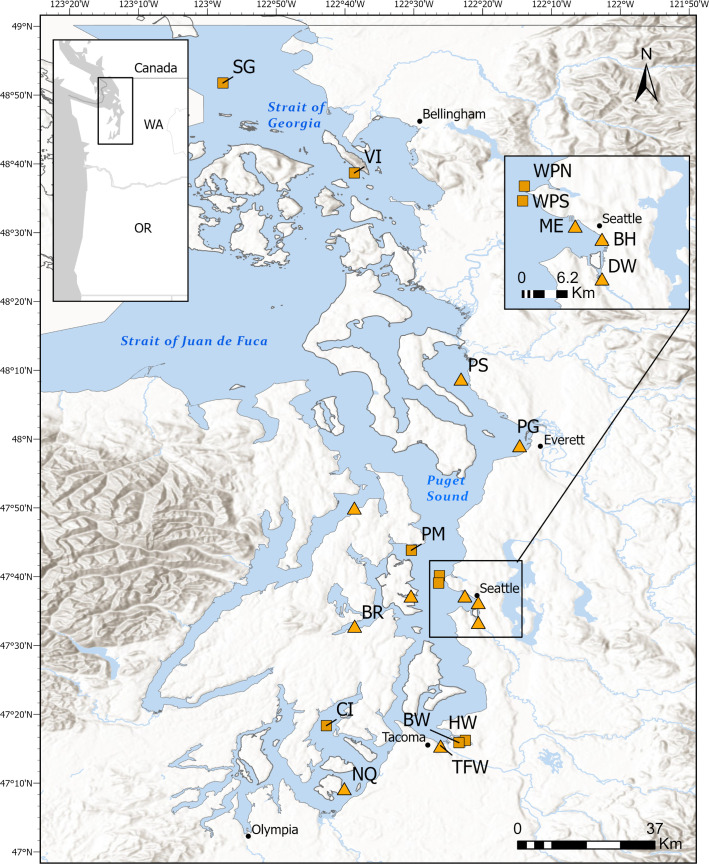
Marine sites sampled by Washington Department of Fish and Wildlife for English Sole bile in the Salish Sea. SG: Strait of Georgia, VI: Vendovi Island, PS: Port Susan, PG: Port Gardner Bay, HC: Hood Canal, PM: Port Madison, WPN: West Point North, WPS: West Point South, EH: Eagle Harbor, ME: Myrtle Edwards, BH: Bell Harbor, DW: Duwamish Waterway, BR: Bremerton, HW: Commencement Bay-Hylebos Waterway, BW: Commencement Bay-Blair Waterway, TFW: Commencement Bay-Thea Foss Waterway, CI: Carr Inlet, NQ: Nisqually. Orange triangles indicate sites with field measures of organism response [53], such as vitellogenin (Vtg) induction in male fish and reproductive maturity of females, and orange squares indicate sites without field measures of organism response (as described below).

Bile samples were collected from male and female English sole by the Washington Department of Fish and Wildlife from 18 locations within the Salish Sea from 2011–2019 (Fig 1). Fish bile samples were analyzed for e-EDCs at the National Oceanic and Atmospheric Administration’s Northwest Fisheries Science Center according to da Silva et al. [50]. Prior to the analysis, samples were deconjugated via enzymatic hydrolysis with β-glucuronidase/sulfatase to obtain the total analyte concentration including the glucuronide- and sulfate-conjugated metabolites [50,51]. Other potential minor conjugation forms, such as glucoside- and glutathione-conjugated e-EDCs, were not evaluated by this method. Each batch of fish bile analysis for e-EDCs were validated based on Sloan et al. [52]. To avoid the confounding presence of endogenous steroidal hormones, female samples were omitted from the EAR-based evaluation of estrogenic activity as they will typically exhibit physiologically relevant levels of steroidal estrogens; this would confound the interpretation of the effects of external steroidal estrogen exposure which was the goal of this study. Relevant data not previously published (S1 Table) was made available in the Dryad repository (https://doi.org/10.5061/dryad.mcvdnck9x).

Estrogenic predictions of chemical mixtures using ToxCast *in vitro* HTS data

The development of a method to assess the estrogenic potential of e-EDCs in mixtures in marine environment included several steps. The first three steps were preparatory, laying the foundation for threshold development, and included ER agonist selection, EAR calculations, and comparison of EARs to EEQs.

### Selection of ER agonist assays in ToxCast for identifying e-EDCs

A total of 18 ER agonist assays were used (S3 Table). Sixteen assays were identified by Judson et al. [23] and two additional assays present in the updated ToxCast database (invitroDBv3.5_database) were identified as ER agonist assays by Maloney et al. [54] (S3 Table). All 18 assays targeted only one specific molecular initiating event [55], the activation of the ER receptor. ER antagonism was excluded from this study because including antagonism could reduce or obscure the activation of the estrogen receptor and limit our ability to identify the estrogenic chemicals.

To distinguish between estrogen agonism and false-positive results caused by assay interference [30,36], the results from the ER agonist pathway model were used to predict the probability that an assay response was associated with the estrogen receptor and not a result of assay interference [23]. The model uses ToxCast data to generate an area-under-the-curve (AUC) which summarizes estrogen receptor activity for each chemical. The AUCs are scaled relative to the highly potent EE2, which is assigned an AUC of 1. In our study, chemicals exhibiting activity in ER agonist assays but having AUC scores ≤ 0.01 were considered non-estrogenic and were excluded from further analysis [23,56]. Twenty chemicals that were detected in the Salish Sea and active in ToxCast ER agonist assays but not evaluated by Judson et al. [23] were manually evaluated based on measured response in ToxCast assays and results from the Collaborative Estrogen Receptor Activity Prediction Project (CERAPP) [56] (S2 Text).

### Exposure-activity ratio (EAR) calculations

EARs (Eq 1) were calculated for each sample and each chemical identified as an e-EDC based on its activity in one or more of the 18 ER agonist assays. The extent of the estrogenic response of an individual e-EDC was quantified by estimating the 5^th^ percentile of the logarithmic ACC (ACC_5_; log µM) across the active ER agonist assays for that e-EDC. Not all e-EDCs have ACC data for all 18 ER agonist assays, therefore the 5^th^ percentile was calculated based on available data and then converted to µg/L. The ACC_5_, has been used as a conservative response measure *in vitro*, and is regarded as a cautious estimate of effects concentrations *in vivo* [57].


$$EAR_{ACC5}=\frac{MeasuredEnvironmentalConcentration\left(\frac{\mu g}{L}\right)}{{ACC}_{5}\left(\frac{\mu g}{L}\right)}$$ (1)

For water and WWTP effluent samples, the measured environmental concentration in Eq 1 was directly measured. For mussel and fish tissue, the measured tissue concentrations were converted to an estimated water concentration using bioconcentration factors (BCFs) predicted with OPERA (OPEn saR App) models available through CompTox or with Burkhard [58] for PFAS compounds, as available (see James et al. [1]). For the six e-EDCs detected in tissue, OPERA-predicted BCFs that were within the CompTox reported global applicability domain were utilized [59,60]. For bile samples, because of the absence of water to bile BCF values for many e-EDCs of interest, the measured concentrations were used directly, and separate bile-based threshold values were calculated as described below.

EAR_ACC5_ values for each chemical in a sample were summed to calculate an EAR_mix_ for each sample (Eq 2), assuming the additivity of effects [61–63].


$$EAR_{mix}={\sum \left(EAR_{ACC5}\right)}_{\left[i\right]}$$ (2)

*i *= the individual chemicals in the mixture that are active in at least one of the selected assays.

Response data from ToxCast assays were extracted using the *ToxEval* R package [64] and *Toxicity Explorer* [65]. Response data were filtered using data quality flags following Corsi et al. [34], which were implemented to limit false positive or negative results [29] (S2 Text). Calculation of the EAR_ACC5_ and EAR_mix_ was carried out using *dplyr* [66] and Excel, and visualizations were created using *ggplot2* [67] and the *ToxEval* R package [64].

### EAR_mix_ and 17β-estradiol equivalency quotients comparisons

To verify the accuracy of EAR_mix_ in estimating the estrogenic activity of chemical mixtures, comparisons were made against EEQs calculated with well-established EEFs from Vega-Morales et al. [9] where the EEFs are scaled relative to E2. For the comparison, EEQ_mix_ and EAR_mix_ were calculated with English sole male fish bile data because this dataset was the most extensive (n = 354) and consistently included analysis of steroidal estrogens.

EEQs were calculated by multiplying the measured concentration of an individual chemical in a bile sample by its chemical-specific EEF (Eq 3). For mixtures, EEQ_mix_ was calculated as the sum of EEQ_[i]_s across all e-EDCs present in a bile sample (Eq 4).


$${EEQ}_{\left[i\right]}={MeasuredEnvironmentalConcentration}_{[i}xEEF_{\left[i\right]}$$ (3)

$${EEQ}_{mix}=\sum {EEQ}_{\left[i\right]}$$ (4)

where *i *= individual chemical information, and EEF is the median estradiol equivalency factor for chemical *i* from Vega-Morales et al [9].

A Spearman’s rank correlation was conducted between the EAR_mix_ and EEQ_mix_ to verify their congruence in capturing estrogenic activity. The $\left\{\frac{EARmix}{EEQmix}\right\}$ ratio was then calculated for each bile sample. The median ratio for all bile samples was used to translate EAR-based thresholds (e-EAR thresholds) from EEQ-based thresholds (see Eq 5).

### Development of e-EAR thresholds

Two sets of e-EAR threshold values, one set for water (water-based thresholds) and one set for bile (bile-based thresholds), were developed based on the no observed effect concentration (NOEC) of 0.005 µg/L E2 and lowest observed effect concentration (LOEC) of 0.025 µg/L E2 for Vtg induction in adult male zebrafish (*Danio* rerio) exposed to E2 [68]. The thresholds for water (NOEC_EAR_ and LOEC_EAR_), were calculated with Eq 5. The thresholds for bile (NOEC_bEAR_ and LOEC_bEAR_) were calculated with Eq 6. The range of available bile-water bioaccumulation factors (BCF_bw_; 4000 [69] – 13000 [70]) for E2 in juvenile rainbow trout (*Oncorhynchus mykiss)* was used, resulting in a range for the bile-based thresholds.


$${LOEC}_{EAR}=LOEC*median\left\{\frac{{EAR}_{mix}}{{EEQ}_{mix}}\right\}$$ (5)

$${LOEC}_{bEAR}={{LOEC}_{EAR}*BCF}_{bw}$$ (6)

For the NOEC_EAR_ and NOEC_bEAR_, the NOEC replaced the LOEC in Eq 5 and 6.

The estrogenic potential of field-collected samples was assessed by comparing their EAR_mix_ values to the relevant e-EAR thresholds, where bile samples were compared to the bile-based thresholds and all other matrices (water, wastewater, and tissue back-calculated to a water concentration) were compared to the water-based thresholds. This sample categorization approach is similar to other studies that have used NOEC and LOEC values to categorize samples into high-, medium-, and low-risk groups using EEQ_mix_ values [71,72]. It is important to emphasize that these thresholds are not intended to be used for regulatory decisions but instead as tools to evaluate the possible estrogenic activity of chemical mixtures.

### Application and qualitative validation of bile-based thresholds for site classification

**Fig 2:**
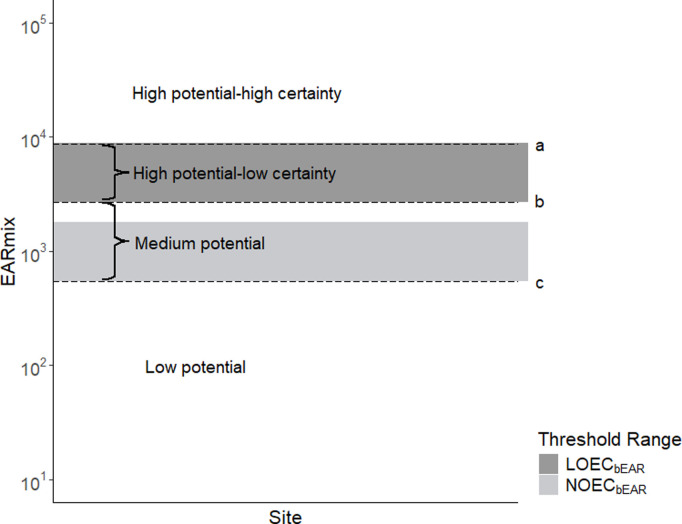
Classification regions for bile sampling sites based on the potential to cause estrogenic effects. Classification was determined by the 95^th^ percentile of EAR_mix_, and calculated for each of 18 sites. Regions are distinguished by the highest LOEC_bEAR_ **(a)**, lowest LOEC_bEAR_ **(b)**, and the lowest NOEC_bEAR_ **(c)**. Classification regions (e.g., low or medium potential for estrogenic effects) are described in the text. Abbreviations: EAR_mix_ (sum of the 5^th^ percentile of the exposure-activity ratios for chemicals in a mixture), LOEC_bEAR_ and NOEC_bEAR_ (bile-based thresholds for estrogenic potential based on the lowest and no observed effect concentrations, respectively).

Bile chemistry data from 18 sites (with n = 6–57 samples/site) were grouped and used to calculate the site-specific 95th percentile of the EAR_mix_ values, representing the upper end of the exposure distribution at each site [73,74]. Sites were classified in the first application of bile-based thresholds by comparing these values with the NOEC_bEAR_ and LOEC_bEAR_ ranges (Fig 2). Specifically, sites with 95th percentile EAR_mix_ values above LOEC_bEAR_ values were considered high potential for estrogenic effects but given the range of available BCF_bw_s for threshold calculations, confidence levels varied depending on the magnitude of exceedance; 95th percentile EAR_mix_ values above the highest LOEC_bEAR_, were classified as high potential with high certainty of estrogenic effects and within the range of LOEC_bEAR_s were high potential with low certainty. The other classifications included medium potential for estrogenic effects when the 95th percentile EAR_mix_ was between the lowest NOEC_bEAR_ and the lowest LOEC_bEAR_; and low potential below the lowest NOEC_bEAR_ (Fig 2).

The bile-based EAR thresholds were qualitatively validated through comparison with field measures of organism response, including percent male Vtg induction, and females with altered reproductive timing (percentage of females in vitellogenic, ripe, or spawning condition outside the normal spawning season). The average percent male Vtg induction and females with altered reproductive timing was compared to the 95^th^ percentile of the EAR_mix_ and the corresponding site classification at each site. EAR_mix_ was considered aligned with measures of organism response when sites with high or medium potential for estrogenic effects corresponded to increased percent Vtg induction or female altered reproductive timing, and lower potential aligned with minimal organism responses. Note that bile samples were collected between 2011 and 2019, while biological observations were collected between 1997 and 2001. To our knowledge, there have not been large scale changes in stressors between those two time periods that would invalidate the comparison. However, we acknowledge the potential that changes we are unaware of may have altered contaminant loadings.

### Application of e-EAR thresholds to prioritize samples and individual chemical constituents in mixtures

EAR_mix_ values calculated for each sample from all sample matrices were compared against the e-EAR thresholds to classify samples according to their estrogenic activity as the second application of the thresholds. Water, WWTP effluent, and tissue samples, were compared to the set of water-based thresholds (NOEC_EAR_ and LOEC_EAR_). For bile samples, the threshold ranges used for site classification were not applied. Instead, the lowest LOEC_bEAR_ and lowest NOEC_bEAR_ were used as a conservative approach and to maintain consistency with the use of water-based thresholds for individual chemical prioritization, since water-based thresholds did not involve a threshold range. For clarity, the term *LOEC threshold* is used to reference both the lowest LOEC_bEAR_ for bile samples and the LOEC_EAR_ for all other sample types, and the *NOEC threshold* refers to the lowest NOEC_bEAR_ for bile samples and the NOEC_EAR_ for all other sample types. Samples were categorized as having low estrogenic activity (EAR_mix_ < NOEC threshold), medium estrogenic activity (LOEC threshold > EAR_mix_ ≥ NOEC threshold), or high estrogenic activity (EAR_mix_ ≥ LOEC threshold). As a sensitivity analysis of how applying the lowest threshold within the bile threshold ranges affected our results, the outcome obtained by utilizing the highest LOEC_bEAR_ in place of the lowest LOEC_bEAR_ was also evaluated.

Samples with medium or high estrogenic activity were further evaluated to prioritize individual chemical constituents for future monitoring and evaluation based on their contribution. Focus was placed on e-EDCs contributing at least 1% towards the mixture response. A chemical was a “driver” of the estrogenic activity if that chemical individually exceeded the threshold; a “major contributor” if it did not individually exceed the threshold but contributed ≥ 1% to the mixture response; or “minor contributor” if it contributed < 1%. A chemical may be categorized as a driver and contributor or assigned to multiple priority categories because different samples may result in conflicting assignments. In cases when chemicals were classified in multiple categories, they were assigned to the higher priority category.

**Fig 3:**
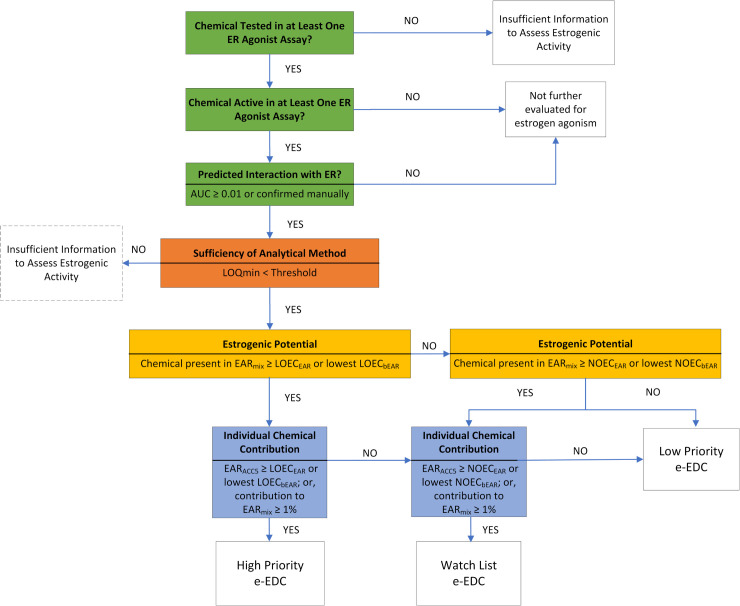
Decision flow diagram for e-EDC chemical constituent prioritization framework. Dotted line around box indicates that this evaluation was not completed for this work (see James et al. [1]). Abbreviations: AUC (area under the curve), LOQ_min_ (minimum level of quantification), EAR_ACC5_ (5^th^ percentile of the exposure-activity ratios for an individual chemical across 18 estrogen receptor agonist assays), EAR_mix_ (sum of EAR_ACC5_ for all chemicals in a mixture), LOEC_EAR_ and NOEC_EAR_ (water-based thresholds for estrogenic potential, corresponding to the lowest observed effect concentration and no observed effect concentration, respectively), and LOEC_bEAR_ are NOECb_EAR_ (bile-based thresholds for estrogenic potential based on the lowest and no observed effect concentration, respectively).

A decision tree for the e-EDC prioritization framework is included (Fig 3). The prioritization categories for e-EDCs are:

- *High Priority*: e-EDCs in mixtures with an EAR_mix _≥ LOEC threshold; and either the EAR_ACC5 _≥ LOEC threshold and is identified as a driver, or the chemical contributes ≥ 1% toward mixture response and is a major contributor. If an individual chemical does not individually exceed the LOEC threshold or contribute ≥ 1% toward the mixture response, but does individually exceed the NOEC threshold, it is a Watch List chemical and a minor contributor.
- *Watch List:* e-EDCs in mixtures with an EAR_mix_ between the NOEC threshold and LOEC threshold; and either the EAR_ACC5 _≥ NOEC threshold and is identified as a driver, or the chemical contributes ≥ 1% toward mixture response and is a major contributor.
- *Low Priority:* e-EDCs in mixtures that were active in ER agonist assays but EAR_mix_ < NOEC threshold or contributed < 1% of mixture response.
- *Insufficient Information:* Insufficient information to assess chemicals as e-EDCs. This category includes CECs that were detected in environmental samples but were not assessed in ToxCast ER agonist assays. Although not evaluated in this work, this also includes chemicals that were analyzed for and not detected. The absence of detection does not preclude the potential to cause estrogenic effects at concentrations below the limit of detection.

## Results

### Identification of e-EDCs using ToxCast ER agonist assay results

Of the 387 chemicals analyzed, 221 were detected in at least one sample (S2 Table). Among the detected chemicals, 157 were evaluated against the ToxCast ER agonist assays; the remaining CECs were either not tested in ToxCast or not tested in the relevant ToxCast assays. Out of those tested in the relevant assays, 68 were active and 89 were inactive (S4 Table). The 68 active chemicals were further evaluated to remove false-positive responses and CECs exhibiting insufficient activity with the ER using either AUC scores or through manual evaluation (S2 Text). This resulted in a final set of 19 active chemicals (Table 1). The 19 e-EDCs were from various chemical classes, as described in James et al. [1] including hormones (n = 9), bisphenols (n = 4), alkylphenols (n = 3), phthalates (n = 2), and one PFAS.

**Table 1:** Chemical classification, minimum and maximum concentrations, and detection frequency (DF) of e-EDCs detected in the Salish Sea.

Chemical Name	CAS	Chemical	Water	Water	WWTP	WWTP	Mussel	Mussel	Fish	Fish	Bile	Bile	Category
	Number	Class	Min – Max (ng/L)	DF	Min – Max (ng/L)	DF	Min – Max (ng/g)	DF	Min – Max (ng/g)	DF	Min – Max (ng/mL)	DF	
17a-Estradiol	57-91-0	Hormones	–	–	1.8 - 1.8	14%	–	–	–	–	–	–	Low Priority
4-n-Octylphenol	1806-26-4	Commercial	–	–	–	–	0.55 - 2.65	64%	0.49 - 2.63	16%	ND	–	Low Priority
4-Nonylphenol	104-40-5	Commercial	13.6 - 41.4	67%	0180 - 1690	33%	10.3 - 34.8	97%	3.12 - 75.6	91%	ND	–	Low Priority
4-tert-Octylphenol	140-66-9	Commercial	–	–	–	–	–	–	–	–	30.0 - 220	15%	Low Priority
Androsterone	53-41-8	Hormones	–	–	0.45 - 0.45	14%	–	–	–	–	–	–	Low Priority
Betamethasone	378-44-9	Hormones	ND	–	–	–	ND	–	3.58 - 3.58	0.6%	–	–	Low Priority
Bisphenol A^1^	80-05-7	Industrial	2.8 - 4.3	3.7%	350 - 6200	56%	3.07 - 4.09	5.5%	2.52 - 40.7	5.1%	4.60 - 810	79%	High Priority
Bisphenol AF	1478-61-1	Industrial	–	–	–	–	–	–	–	–	0.76 - 0.76	0.4%	Low Priority
Bisphenol F	620-92-8	Industrial	–	–	–	–	–	–	–	–	4.60 - 88.0	5.2%	Low Priority
Bisphenol S	80-09-1	Industrial	0.44 - 13.6	100%	–	–	–	–	–	–	0.76 - 30.0	37%	Low Priority
Butyl benzyl phthalate	85-68-7	Phthalates	–	–	1700 − 1700	14%	–	–	–	–	–	–	Low Priority
Desogestrel	54024-22-5	Hormones	–	–	1.01 - 7.04	29%	–	–	–	–	–	–	Low Priority
Dibutyl phthalate	84-74-2	Phthalates	–	–	920 − 920	14%	–	–	–	–	–	–	Low Priority
Estradiol^2^	50-28-2	Hormones	–	–	11.9 - 11.9	14%	–	–	–	–	0.99 - 2700	93%	High Priority
Estriol^3^	50-27-1	Hormones	–	–	ND	–	–	–	–	–	0.86 - 540	26%	High Priority
Estrone^4^	53-16-7	Hormones	–	–	20.2 - 1000	29%	–	–	–	–	0.96 - 1600	100%	High Priority
Perfluorooctanesulfonamide	754-91-6	PFAS	ND	–	ND	–	0.62 - 0.62	5.6%	0.10 - 3.38	47%	–	–	Low Priority
Prednisone	53-03-2	Hormones	ND	–	–	–	ND	–	26.9 - 26.9	0.6%	–	–	Low Priority
Trenbolone acetate	10161-34-9	Hormones	ND	–	–	–	ND	–	0.23 - 0.31	1.3%	–	–	Low Priority

“ND” is non-detect and “-” indicates that the chemical was not analyzed in that matrix.

“Category” was determined from this work; determination of these categories and footnotes 1–4 will be further discussed in the “Categorization of sample mixtures and prioritization of chemical constituents” section.

^1^*High Priority* based on bile and wastewater treatment plant (WWTP) effluent. Bisphenol A (BPA) was a contributor in samples classified as high potential for estrogenic effects based on bile and WWTP effluent. In some WWTP effluent samples, BPA was also a driver in samples classified as medium potential for estrogenic effects.

^2^*High Priority* based on bile and WWTP effluent. Estradiol (E2) was a contributor to samples classified as high potential for estrogenic effects based on WWTP effluent and was a driver based on bile.

^3^*High Priority* based on bile. Estriol (E3) was a driver in samples classified as high potential for estrogenic effects based on bile.

^4^*High Priority* based on bile and WWTP effluent. Estrone (E1) was a driver in samples classified as high potential for estrogenic effects based on bile and WWTP effluent

### Chemical mixtures and the occurrence and distribution of e-EDCs in environmental monitoring samples

The monitoring data demonstrates the presence of complex mixtures of CECs in the Salish Sea. Individual samples contained as many as 55 unique chemicals in a mixture (e.g., in a WWTP effluent sample), with 49 of these chemicals not identified as e-EDCs based on the measurement and activity in the 18 ER assays (S5 Table). From each matrix, the maximum number of e-EDCs detected together in a sample were two in marine water, six in WWTP effluent, three in mussel tissue, four in fish tissue, and six in fish bile (S5 Table). Due to the opportunistic nature of including datasets from several monitoring campaigns, the analysis of e-EDCs varied across sampling matrices. For example, the analysis of steroidal estrogens was limited to fish bile and WWTP effluent samples. The detection frequency of e-EDCs also varied across matrices. The 19 e-EDCs that were detected, their concentration ranges, and corresponding detection frequencies for each sample matrix are presented in Table 1.

In addition to different matrices, samples were collected from different aquatic species. Biological samples from either whole body or edible filet tissues were collected from eight fish species and one shellfish species, representing different diets, habitats, and thus, varying exposure profiles. The variation in chemical occurrence and concentration across different species is included in S6 Table.

### EEQ_mix_ to EAR_mix_ comparison and determination of e-EAR thresholds

EEQ_mix_ and EAR_mix_ values were calculated using chemistry data for 354 male bile samples. The values were strongly correlated (Spearman’s rank correlation coefficient of ρ = 0.98 (p < 0.01, n = 354;S1 Fig)). Of the chemicals detected in samples from this study, the ToxCast EAR_mix_ approach identified 19 as e-EDCs compared to seven, using the EEF approach [7].

e-EAR thresholds were determined based on the relationship between EEQ_mix_ and EAR_mix_. Using the five e-EDCs detected in bile that had an EEF, the $median\left\{\frac{EARmix}{EEQmix}\right\}$ ratio was 27 (S2 Fig). This was used to calculate a NOEC_EAR_ and LOEC_EAR_ of 0.14 and 0.68, respectively. The bile-based thresholds were estimated by applying the range of E2 BCF_bw_ values to the water-based thresholds resulting in a range of NOEC_bEAR_ values from 540 to 1800; and LOEC_bEAR_ values from 2700 to 8800. These ranges are depicted in Fig 2.

### Classification of sampling locations using bile data and validation of e-EAR thresholds

**Fig 4:**
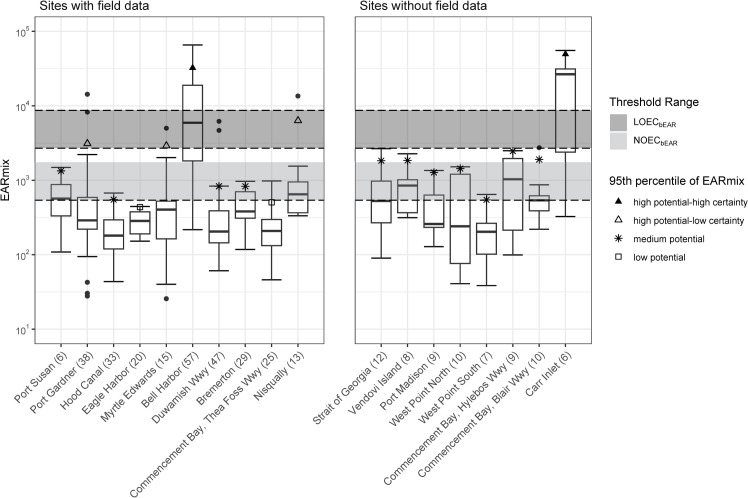
Distribution of EAR_mix_ values for sites with field measures of organism response from Johnson et al. [53], and sites without field data. Site classification is based on regions which are determined by the highest LOEC_bEAR_, lowest LOEC_bEAR_, and lowest NOEC_bEAR_, indicated by dashed lines. Box and whisker plot shows the median, first/third quartile, and a distance of 1.5 times the interquartile range. Data outside of the box and whisker plot are marked with a closed circle. The remaining symbols represent the 95^th^ percentile of the EAR_mix,_ with each symbol representing the corresponding categorization. The number in parenthesis represents the number of samples collected at that site. Abbreviations: EAR_mix_ (sum of the 5^th^ percentile of the exposure-activity ratios for chemicals in a mixture), LOEC_bEAR_ and NOEC_bEAR_ (bile-based thresholds for estrogenic potential based on the lowest and no observed effect concentrations, respectively), Wwy (waterway).

All 18 bile sampling locations, including the eight sites that did not have field measures of organism response for the validation exercise, were classified for potential estrogenic effects using the 95^th^ percentile EAR_mix_ (Fig 4). Two sites (Eagle Harbor and Commencement Bay-Thea Foss Waterway) were below the lowest NOEC_bEAR_ and were classified as low potential for estrogenic effects; 11 sites (Port Susan, Hood Canal, Duwamish Waterway, Bremerton, Strait of Georgia, Vendovi Island, Port Madison, West Point North, West Point South, Commencement Bay-Hylebos Waterway, and Commencement Bay-Blair Waterway) were considered medium potential; three (Port Gardner, Myrtle Edwards, and Nisqually) were characterized as high potential-low certainty; and two sites (Bell Harbor and Carr Inlet) were considered high potential-high certainty based on EAR_mix_ (Fig 4). Summary statistics of EAR_mix_ for all 18 bile sampling locations can be found in supplemental information S7 Table.

Overall, comparisons between the 95^th^ percentile of the EAR_mix_ and at least one field measure of organism response were consistent, validating the e-EAR thresholds and the use of EAR_mix_ as a measure of endocrine disruption. For example, there was alignment observed at 8 of 10 sites: three of four high potential sites had the among the highest percentage of male Vtg induction, all four medium potential sites had intermediate percentages of male Vtg induction, and one of the two low potential sites had no males expressing Vtg. Additionally, two high potential sites had high percentages of females with altered reproductive timing (Table 2).

**Table 2:** Heatmap illustrating the classification of sample sites with field measures of organism response.

Site ID (number of samples for: EARmix/ % Male Vtg Induction/ % Female spawning or vitellogenic/ripe)	EAR_mix_ (95^th^ percentile)	% Male Vtg induction	% Females spawning or vitellogenic/ripe
Port Susan (6/44/40)	1338	6.8	8
Port Gardner (38/16/214)	3119	18.8	9
Hood Canal (33/35/235)	548	5.7	6
Eagle Harbor (20/25/138)	436	0	4
Myrtle Edwards (15/49/21)	2913	46.9	81
Bell Harbor (57/32/107)	32346	37.5	65
Duwamish Wwy (47/17/25)	831	17.6	0
Bremerton (29/17/95)	830	5.9	6
Commencement Bay-Thea Foss Wwy (25/23/146)	505	21.7	2
Nisqually (13/17/222)	6345	0	5
Strait of Georgia (12/NA/NA)	1831	NA	NA
Vendovi Island (8/NA/NA)	1848	NA	NA
Port Madison (9/NA/NA)	1273	NA	NA
West Point North (10/NA/NA)	1436	NA	NA
West Point South (7/NA/NA)	546	NA	NA
Commencement Bay-Hylebos Wwy (9/NA/NA)	2466	NA	NA
Commencement Bay-Blair Wwy (10/NA/NA)	1901	NA	NA
Carr Inlet (6/NA/NA)	49519	NA	NA

Blue: Low potential for estrogenic effects; yellow: Medium potential for estrogenic effects; Orange: High potential, low certainty of estrogenic effects; Red: High potential, high certainty of estrogenic effects.

Classification is based on the 95^th^ percentile of the EAR_mix_, using male English sole bile samples collected from 2011 to 2019. Field measures of organism response include the percentage of males expressing vitellogenin (% male Vtg induction) and the percentage of females exhibiting altered reproductive timing (% females spawning or vitellogenic/ripe) surveyed in English sole between 1997 and 2001 [53]. NA indicates that the site was not included in Johnson et al. [53]. Abbreviations: EAR_mix_ (sum of the 5^th^ percentile of the exposure-activity ratios for chemicals in a mixture), Vtg (vitellogenin), Wwy (waterway).

### Categorization of sample mixtures and prioritization of chemical constituents

**Fig 5:**
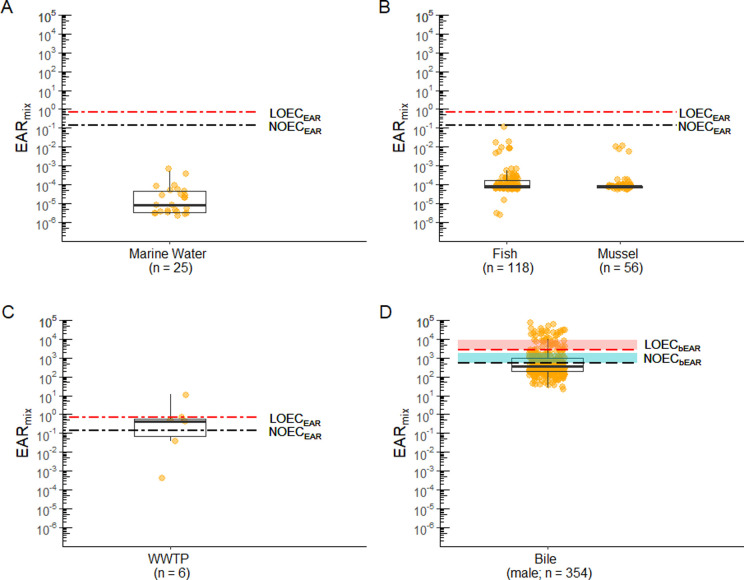
Distribution of EAR_mix_ values for each matrix: (A) marine water, (B) tissues (C) WWTP effluent, and (D) fish bile. Box and whisker plot shows the median, first/third quartile, and a distance of 1.5 times the interquartile range. The closed circles represent the EAR_mix_ values for each sample, and n is the number of samples with detected e-EDCs. For tissues samples **(B)**, concentrations were back-calculated to water concentrations; steroidal estrogens were not included in the analysis of fish and mussel tissue or marine water. NOEC_EAR_ and LOEC_EAR_ thresholds for marine water, tissues, and WWTP effluent are represented by dotted and dashed lines (A, B, **C)**. The lowest NOEC_bEAR_ and lowest LOEC_bEAR_ thresholds for bile are represented by dashed lines, and the threshold ranges for bile are represented by the shaded regions **(D)**. Abbreviations: EAR_mix_ (sum of the 5^th^ percentile of the exposure-activity ratios for chemicals in a mixture), LOEC_EAR_ and NOEC_EAR_ (water-based thresholds for estrogenic potential, corresponding to the lowest observed effect concentration and no observed effect concentration, respectively), and LOEC_bEAR_ are NOECb_EAR_ (bile-based thresholds for estrogenic potential based on the lowest and no observed effect concentration, respectively), WWTP (wastewater treatment plant).

In the second application of the e-EAR thresholds, the estrogenic activity of all sample mixtures was predicted irrespective of sample collection location. None of the e-EDC mixtures measured in marine water or tissue samples exceeded the water-based thresholds, suggesting low estrogenic activity from the chemicals considered in this investigation (Figs 5A and 5B). However, steroidal estrogens, which are important drivers of estrogenic activity, were not analyzed in marine water and tissue samples, so it is possible that estrogenic activity was underestimated in these samples.

Among the nine WWTP effluent samples, which represented a worst-case exposure scenario, six had detectable e-EDCs. One sample exceeded the LOEC_EAR_ of 0.68 (high estrogenic activity) and three samples exceeded the NOEC_EAR_ of 0.14 (medium estrogenic activity) (Fig 5C).

Of the 354 bile samples from male English sole, 47 (13%) had EAR_mix_ values above the lowest LOEC_bEAR_ (EAR_mix_ ≥ 2700) and were classified as high estrogenic activity (Fig 5D). Eighty-six (24%) had EAR_mix_ values between the lowest LOEC_bEAR_ and lowest NOEC_bEAR_ (540 ≤ EAR_mix_ < 2700) and were classified as medium estrogenic activity (Fig 5D).

### Prioritization of chemical constituents based on measurements in WWTP effluent

**Fig 6:**
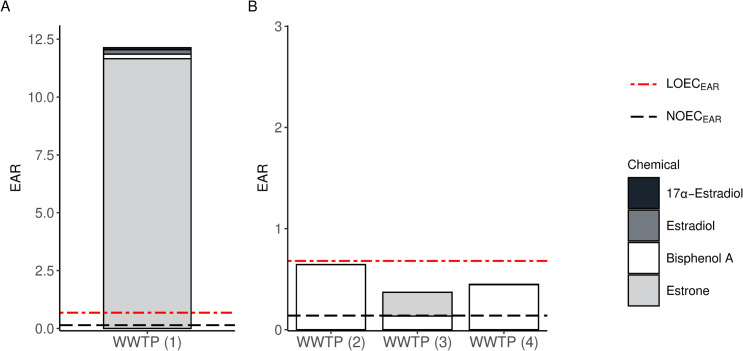
Stacked bar plot with exposure-activity ratios (EARs) for wastewater treatment plant (WWTP) effluent samples exceeding the LOEC_EAR_ (A) or NOECEAR (B). The EAR_ACC5_ for each chemical and sum of the EAR_ACC5_ (EAR_mix_) for the mixture are shown. The dotted and dashed line is the LOEC_EAR_, and the dashed line is the NOEC_EAR_. Minor contributions from androsterone, desogestrel, butyl benzyl phthalate, dibutyl phthalate, and 4-nonylphenol are not visualized because the values are too small to be discernible. WWTP are anonymized sites in Puget Sound represented by 1-4. Abbreviations: EAR_ACC_ (the 5^th^ percentile of the activity cutoff concentrations from ER agonist assays), EAR_mix_ (sum of the EAR_ACC_ for chemicals in a mixture), LOEC_EAR_ and NOEC_EAR_ (water-based thresholds for estrogenic potential, corresponding to the lowest observed effect concentration and no observed effect concentration, respectively).

The chemicals responsible for the four WWTP effluent sample exceedances are presented in Fig 6. E1, E2, and BPA were all important in driving or contributing to estrogenic response and so identified as *High Priority*. There were minor contributions (<1%) from androsterone, desogestrel, 17α-estradiol, butyl benzyl phthalate, dibutyl phthalate, and 4-nonylphenol in selected samples.

### Prioritization of chemical constituents based on measurements in bile

Based on the lowest LOEC_bEAR_ exceedances in bile samples, E1, E2, E3, and BPA were classified as *High Priority* e-EDCs. One or more of these chemicals were identified in 47 bile samples with an EAR_mix_ ≥ lowest LOEC_bEAR_ and classified as high estrogenic activity (S3 Fig). Eighty-seven percent of samples with high estrogenic activity had at least one driver, with 34% of those samples having one, 59% having two, and 7% having three drivers. In all cases, the driver(s) were steroidal estrogens (E1, E2, and/or E3). BPA was a major contributor in one of the 47 samples. As a sensitivity analysis, a less conservative evaluation using the highest LOEC_bEAR_ as the threshold (EAR_mix_ ≥ 8800) resulted in 28 samples exceeding the LOEC_bEAR_, with 23 samples having at least one driver. In this scenario, based on the bile samples, E3 would be downgraded to a contributor and BPA would no longer be a major contributor, and so not a *High Priority* e-EDC.

An additional 86 bile samples had an EAR_mix_ between the lowest NOEC_bEAR_ and lowest LOEC_bEAR_, suggesting medium estrogenic activity (S4 Fig). E1 or E2 were the only drivers; E3 and BPA were major contributors. There were minor contributions (<1%) from 4-tert-octylphenol, bisphenol AF, bisphenol F, and bisphenol S in select samples identified with high and medium estrogenic activity.

Overall, in 51% of bile and WWTP effluent samples above the NOEC threshold and 88% above the LOEC threshold, the mixture surpassed the threshold because of a single chemical alone rather than a mixture of chemicals.

## Discussion

### EAR_mix_ as a measure of estrogenic activity

Expanding the utilization of ToxCast data to assess chemical mixtures with a common mode of action was a key focus of this study. To meet this objective, the use of EAR_mix_ as a measure of estrogenic activity was first confirmed based on a comparison with the well-established EEQ_mix_ [7,9,75,76]. The results demonstrated a strong correlation between EAR_mix_ and EEQ_mix_, supporting the use of EAR_mix_ as a metric for assessing estrogenic activity. The advantage in using EAR_mix_ compared to EEQ_mix_ is that it integrates data from multiple assays measuring various endpoints along the endocrine signaling pathway and EARs are available for more chemicals compared to EEQs. These considerations underscore the benefits of using EAR_mix_ derived from *in vitro* programs, particularly since live animal testing is becoming more restricted [77,78].

### Limitations and considerations for assessing estrogenic activity of mixtures

There are several potential considerations when estimating estrogenic activity of environmental samples. First, not all potential e-EDCs were measured in each sample or sample matrix evaluated in this work. For example, steroidal estrogens were not measured in any of the marine water, mussel, or fish tissue samples. This is in part because steroidal estrogens commonly occur below analytical detection limits in these matrices. Since the majority of estrogenic activity is attributable to steroidal estrogens [7,79,80], assessments where steroidal estrogen data is lacking would underestimate total estrogenic activity. Future work should also consider the challenge of distinguishing endogenous estrogen levels from exogenous exposure, as with any naturally occurring compound.

Additionally, the scope of evaluation is limited to those chemicals currently represented in ToxCast. For example, legacy contaminants such as polychlorinated biphenyls, hydroxylated-polycyclic aromatic hydrocarbons, and polybrominated diphenyl ethers, which have been documented in tissue samples from the region [81,82] and are weakly estrogenic [83,84], were not included because they remain largely untested in ToxCast [85]. Consequently, it is probable that legacy pollutants are present and actively contribute toward estrogenic activity in marine species (e.g., Commencement Bay–Thea Foss Waterway).

This study prioritized e-EDCs based on their direct agonism of the ER, as measured in the selected ER agonist assays from ToxCast. Consequently, this methodology does not capture chemicals that indirectly affect estrogen receptor signaling through alternative modes of action. For example, trenbolone acetate, a *Low Priority* e-EDC, is an androgen that is fed to livestock to promote growth and can enter the aquatic environment through agricultural runoff [86]. The metabolite, 17β-trenbolone, acts as a weak ER agonist, but can also antagonistically impact estrogen signaling [23] by accelerating E2 clearance, leading to decreased endogenous E2 levels in female fish [87]. Additionally, chemicals that do not individually act like estrogen agonists may synergistically amplify the effects of E2 on the ER signaling pathway. For instance, the ToxCast data indicates that perfluorooctane sulfonate (PFOS) and perfluorooctanoic acid (PFOA) are not direct estrogen agonists. Sonthithai et al. [88] similarly found no *in vitro* estrogenic activity individually for PFOS or PFOA; however, co-exposure with E2 resulted in enhanced effects of E2, demonstrating that chemicals need not directly interact with the ER to influence the ER signaling pathway.

With respect to site-level classification, sample size may be a limitation that influenced results. For example, at the Nisqually site, bile samples were obtained from only 13 fish, and the 95^th^ percentile of the EAR_mix_ was disproportionally influenced by a single sample (Fig 4).

### Bile-based threshold development and validation

Another objective of this work was to develop and validate meaningful threshold values for measurements of chemical concentrations in bile. Many e-EDCs accumulate in bile, which provides an opportunity to characterize exposures that are not detectable in other tissues [47]. For example, exogenous estrogens tend to concentrate as glucuronide and sulfate conjugates in fish bile [45,69,89], and their total concentration (i.e., free and conjugated) measured in fish bile is an effective way to characterize recent environmental exposures [46,90–92]. However, in order to interpret the significance of chemical concentrations in bile, tissue-specific thresholds are needed. These were developed in this study.

The development of e-EAR thresholds was based on traditional measures of toxicity (i.e., NOECs and LOECs; used as benchmarks for endocrine disruption [71]). Despite recognized limitations of NOECs and LOECs [93–95], they remain a practical choice due to their availability and application in similar studies [71,72,96]. To validate bile-based thresholds, measures of e-EDC exposure in an organism, percent male Vtg induction and percent female altered reproductive timing, were used.

EAR_mix_ along with percent male Vtg induction and percent female altered reproductive timing serve as distinct lines of evidence reflecting e-EDC exposure, capturing information across varying timescales. e-EDC concentrations in bile reflect recent environmental exposures. Therefore, EAR_mix_, derived from measured biliary e-EDC concentrations, is sensitive to rapid biliary turnover, making it reflective of recent exposure and so is a temporally dynamic measure [91,97,98]. Vtg induction in males is also temporal with detectable presence within a few days of exposure and can persist in the bloodstream for months [5,99]. Vtg induction is commonly used as a bioindicator of exposure and a warning sign [100] for more significant estrogenic effects, like altered reproductive timing, reduced fertility or fecundity, and intersex gonads [9,68]. Both Vtg induction and EAR_mix_ offer insights primarily into episodic exposures, whereas changes in female reproductive timing may indicate prolonged or chronic exposures.

For the validation, a qualitative assessment was used rather than statistical analysis because the field measures used for validation were collected during a different time period. Considering the time difference between data collection periods, perfect alignment was not expected; however, 8 of 10 sites showed reasonably close agreement. Specifically, the site classified as high potential-high certainty, Bell Harbor, exhibited higher proportions of males with Vtg induction and females with altered reproductive timing, while sites classified as medium potential showed notably lower proportions, and sites with low potential had minimal evidence of estrogenic effects from field observations.

For the eight sites lacking field measured data, we recommend further investigation and data collection, particularly at Carr Inlet, which has the highest 95th percentile of the EAR_mix_. Carr Inlet is located in a low-development area with minimal impervious surfaces and the absence of known point sources, yet it likely receives non-point source wastewater inputs via on-site sewage systems which could explain the elevated exposures along with other hydrodynamic factors. Given the complex geography and bathymetry of the Salish Sea, wastewater treatment plant effluent distribution is influenced by factors such as stratification and the hydraulic residence time in embayments. As such, WWTP-associated contaminants such as E1 and E2 may be present at relatively high concentrations in less-urbanized embayments that otherwise receive little direct wastewater inputs. Field measures of exogenous exposure at Carr Inlet would help validate the high EA_Rmix_ and support source investigation.

### High priority and watch list e-EDCs

Four CECs (E1, E2, E3, and BPA) were identified as *High Priority* e-EDCs in the current study. In James et al. [1], BPA was identified as *High Priority* based on its presence in WWTP effluent and fish tissue, and E1 and E2 were deemed *High Priority* based on their presence in undiluted WWTP effluent alone (i.e., as a worst-case scenario). In this study, E1 and E2 were prioritized based on their presence in both WWTP effluent and bile samples, incorporating a biologically relevant measure and removing the worst-case scenario designation based solely on WWTP effluent concentrations. Additionally, the inclusion of bile chemistry data enabled the evaluation of previously undetected chemicals like E3, also prioritized as a *High Priority* chemical in this study. E1, E2, and E3 were identified as individual drivers in the majority of bile samples exceeding the thresholds. Of the bile samples exceeding the LOEC_bEAR,_ 57% included contributions to the EAR_mix_ from multiple steroidal estrogens. This suggests that mixtures of steroidal estrogens are commonly responsible for the estrogenic activity, emphasizing the need to further explore potential effects of these e-EDCs in combination.

BPA was the only CEC included as a *High Priority* e-EDC because of its contribution toward mixture response rather than as an individual driver. BPA is a high production volume chemical and a common component in many industrial products, spanning from clothing to food industry [101]. It is widespread in the natural environment, and found in surface water, effluent discharges, and wildlife [101]. In this study, BPA was present in marine water, WWTP effluent, mussel and fish tissue, and fish bile (Table 1). BPA was present at higher concentrations and detection frequencies in fish bile than whole body/fillet fish tissue, aligning with previous findings that BPA and alkylphenols concentrate in fish bile [47,48,89]. These results underscore the value of utilizing fish bile to assess e-EDC exposures. Moreover, a recent review highlighted the versatility of fish bile data as a biomarker for environmental pollutants, extending beyond estrogenic chemicals to include contaminants such as PAHs and their metabolites, metals, pesticides, pharmaceuticals, resin acids, hepatotoxins, and PFAS [91].

In the second application of the e-EAR thresholds, the prioritization of individual chemicals measured in bile relied upon two bile-based thresholds (i.e., the lowest NOEC_bEAR_ and LOEC_bEAR_), as opposed to three thresholds (i.e., highest LOEC_bEAR,_ lowest LOEC_bEAR_, lowest NOEC_bEAR_) and resulting ranges used for site classification (Fig 2). Two thresholds were selected for individual chemical prioritization to maintain consistency with the water-based prioritization method. Opting for the lowest threshold values within the ranges proved more sensitive, with E3 upgraded to a driver rather than contributor, and the *High Priority* classification of BPA supported with a marine sample rather than just WWTP effluent. This choice of the most conservative threshold aligns with the study’s objectives, as the results are not intended for regulatory action but rather for focusing future investigations.

### Low priority and insufficient information chemicals

Fifteen e-EDCs were categorized as *Low Priority* because they contributed less than 1% toward estrogenic response in mixtures that exceeded the threshold (Table 1). This is caused by low concentrations and/or low potency, compared to the *High Priority* chemicals. For instance, 17α-Estradiol (αE2), a stereoisomer of E2, is illustrative of a *Low Priority* e-EDC with high potency and low measured environmental concentrations. In the ER model, αE2 has a higher AUC (1.06) than E2 (0.94), indicating a greater likelihood to interact with the estrogen receptor [23]. Additionally, the absence of bile data for αE2 represents a gap in chemistry data that may have impacted its categorization [102]. Given the lack of commercially available analysis of αE2 in biological samples, the increased development and availability of methods for its quantification are recommended.

The 14 *Low Priority* e-EDCs with lower potencies than αE2 and E2 based on Judson et al. [23] are alkylphenols (e.g., octylphenol and 4-nonylphenol), alternative bisphenols (e.g., bisphenol S, bisphenol F, and bisphenol AF), phthalates, PFOSA, and other classes of hormones besides estrogens. The estrogenic potency of these chemicals, as measured by ACC5 values, ranged between 86 and 200,000 times lower than E2 (S4 Table). Although results suggest that the Low Priority e-EDCs are not drivers or major contributors toward ER agonism, they may exert effects through alternative modes of action. For instance, betamethasone and prednisone are synthetic corticosteroids frequently used to treat inflammatory and immune diseases in human and veterinary medicine [103]. They primarily act through glucocorticoid receptor agonism [103]. Future work should consider additional modes of action beyond ER agonism. based on the methods outlined here.

In some instances, chemicals are a lower priority here than in James et al. [1] where a single mode of action was not the focus. In the current work, betamethasone, prednisone, PFOSA, and butyl benzyl phthalate were categorized as *Low Priority* e-EDCs, whereas they were previously prioritized as *High Priority* CECs [1]. Similarly, αE2, 4-nonylphenol, dibutyl phthalate, and trenbolone acetate, were classified as *Low Priority* e-EDCs, but were *Watch List* CECs in James et al. [1]. This difference in categorization can be attributed to the ability of chemicals to elicit biological responses through multiple modes of action as accounted for in James et al. [1]. In cases where different prioritization approaches result in conflicting classifications, our recommendation is to retain these chemicals in the highest prioritization category. For a current list of recommended CEC prioritizations see the Figshare repository (https://doi.org/10.6084/m9.figshare.26195573).

CECs in the *Insufficient Information* category were not evaluated because data associated with either their potential to interact with the ER pathway or their environmental occurrence was lacking. This included 61 chemicals that have not been evaluated with ToxCast ER agonist assays (S8 Table). As more bioactivity information becomes available, these 61 chemicals should be re-evaluated. There were also CECs that were active in ER agonist assays but were not detected in the environment above the limit of quantification (LOQ); these may be of concern at levels below the LOQ. One such chemical is EE2, one of the most potent estrogenic chemicals [104], which was analyzed but not detected above the LOQ in WWTP effluent or bile. Recent monitoring of EE2 in marine water had an LOQ of approximately 5 ng/L [105], which would result in an EAR_ACC5_ greater than the NOEC_EAR_ threshold, suggesting that it could contribute to an estrogenic response. Therefore, continued improvements to analytical techniques are necessary, and chemicals like EE2 and transformation products should continue to be monitored in the environment.

## Conclusion

This study developed a novel approach to evaluate the estrogenic activity of CEC mixtures in various environmental sample types collected from the Washington State Salish Sea, including fish bile. Estrogenic activity was assessed using *in vitro* HTS data from ToxCast and EAR_mix_ calculations, which aligned with traditional measures of estrogenicity. New e-EAR thresholds were established for mixtures in water and fish bile, enabling the biological interpretation of fish bile concentrations. Qualitative validation of thresholds with field measures, such as Vtg induction and altered reproductive timing, supported the utility of bile-based thresholds and highlighted the importance of prioritizing areas with high-potential for estrogenic effects like Carr Inlet for further investigation. This study also demonstrated that a small number of chemicals, particularly natural estrogens (E1, E2, and E3) contribute most to estrogenic activity. Wastewater is a primary pathway by which these natural estrogens enter the aquatic environment, so alterations to WWTP [106] and on-site sewage system [107] operations and infrastructure may be one approach to reduce their environmental impacts. Considering the transformation of E1, E2, and E3 in the environment and within organisms [63,108–111], managing these steroidal estrogens collectively is recommended. Overall, this study strengthened regional prioritization efforts, used to inform environmental management decisions and future investigations. This work also demonstrated the broader applicability of *in vitro* HTS data for evaluating estrogenic activity in chemical mixtures across multiple matrices, offering a versatile methodology that can be adapted to other ecosystems, matrices, and modes of action.

## Supporting information

S1 Table Summary of sampling campaigns that were included in this study. (DOCX)

S1 Text Data QAQC Review Notes. (DOCX)

S2 Table Analysis and detection counts of all CECs in water, WWTP effluent, mussel tissue, fish tissue, and English sole bile. (XLSX)

S3 Table Summary of ToxCast assays related to the estrogen receptor agonist signaling pathway. Unless stated otherwise, ER agonists assays were identified by Judson et al. (2015).
(DOCX)

S2 Text ER Agonist manual evaluation. (DOCX)

S4 Table ER agonist determinations. (XLSX)

S5 Table Mixture complexity of Salish Sea samples. (XLSX)

S6 Table Median, minimum, and maximum chemical concentrations (ng/g) per e-EDC per species and tissue type. (XLSX)

S1 Fig Scatterplot and Spearman’s rank correlation coefficient between EEQ and EARA_CC5_ calculated for male fish bile samples (n = 354). (TIF)

S2 Fig Density plot of the distribution of EAR_mix_:EEQ_mix_ values. (TIF)

S7 Table EAR summary statistics for each bile sampling site. (XLSX)

S3 Fig Stacked bar plot with exposure-activity ratios (EARs) for each bile sample with EARs exceeding the LOEC_EAR._ Stacked bar plot with exposure-activity ratios (EARs) for each bile sample with EARs exceeding the LOEC_bEAR_, represented by the red dashed line. The sample labels include the site ID abbreviation (as described in Fig 1), collection year, and a letter to distinguish samples. The EAR_ACC5_ for each chemical and the overall EAR_mix_ for the sample are shown. The dotted and dashed line represents the lowest NOEC_bEAR,_ and the dashed line represents the lowest LOEC_bEAR_ calculated using the lowest BCF_bw_. The shaded regions represent the range of calculated possible threshold values based on range of BCF_bw_ values.
(TIF)

S4 Fig Stacked bar plot with exposure-activity rations (EARs) for each bile sample with EARs between the NOEC_EAR_ and LOEC_EAR._ Stacked bar plot with exposure-activity ratios (EARs) for each bile sample with EARs between the LOEC_bEAR_ and the NOEC_bEAR_, represented by the black dashed line. The sample labels include the site ID abbreviation (as described in Fig 1), collection year, and a letter to distinguish samples. The EAR_ACC5_ for each chemical and the overall EAR_mix_ for the sample are shown. The the shaded region represents the range of calculated possible NOEC threshold values based on range of BCF_bw_ values.
(TIF)

S8 Table Chemicals with insufficient information. (XLSX)
