# Supplementary material for: Estrogenic activity of mixtures in the Salish Sea: The use of high throughput toxicity data with chemical information from fish bile and other matrices
Source: PLoS One. 2025 Jul 9;20(7):epone.0323865. doi: 10.1371/journal.pone.0323865 (PMC12240389; doi:10.1371/journal.pone.0323865)
Supplement: S1 Table — (DOCX) [file pone.0323865.s001.docx]

**S1 Table. Summary of sampling campaigns that were included in this study**. Study names marked with an asterisk (*) were used to complete the estrogenic-endocrine disrupting (e-EDC) chemical mixture evaluation. For these studies, datasets that were not previously published before this work were made available through a data repository, as indicated in “Data Availability” column and located here: [https://doi.org/10.5061/dryad.mcvdnck9x](https://urldefense.com/v3/__https:/doi.org/10.5061/dryad.mcvdnck9x__;!!K-Hz7m0Vt54!mFX5Y3TrGIfV06-vM3yOdv7NZJNXq9OEc5sCOca3vcO-s9i59_QP_hsVKvymZY38tCjA7zeMBrhYg_1xbgkkaaosalql$). For the other datasets sourced from third parties, the relevant data can be accessed either through the reports themselves or within their supplemental materials, as specified in the “Data Availability” column. Studies that were evaluated but not used in the e-EDC mixture evaluation are labeled as not applicable (NA) in the last column. Studies requiring permits for sample collection are indicated in the “Reference” column^1^.

| **Reference** | **Study Name** | **Sample Year** | **Matrix** | **Sample type** | **Tissue type** | **Analytical Laboratory** | **Data Availability** |
| --- | --- | --- | --- | --- | --- | --- | --- |
| Jack and Grassley (2017) | Water Quality Assessment and Monitoring Study: Contaminants of Emerging Concern | 2017 | Water |  |  | SGS-AXYS | NA |
| Lanksbury (2022) | Chemicals of Concern in Marine and Freshwater Fish in King County* | 2021 | Tissue | whole | *Micropterus dolomieu* | SGS-AXYS | Original report or supplemental material |
|  |  |  |  |  | *Sebastes auriculatus* | SGS-AXYS |  |
|  |  |  |  |  | *Sebastes maliger* | SGS-AXYS |  |
|  |  |  |  |  | *Mytilus edulis* | SGS-AXYS |  |
| Meador et al. 2016 | Contaminants of Emerging Concern in a Large Temperate Estuary* | 2014 | Water | estuary and WWTP effluent |  | SGS-AXYS | Original report or supplemental material |
|  |  |  | Tissue | whole | *Oncorhynchus tshawytscha* | SGS-AXYS |  |
|  |  |  | Tissue | whole | *Leptocottus armatus* | SGS-AXYS |  |
| Tian et al. 2020 | Suspect and nontarget screening for CEC in an urban estuary* | 2018 | Water | estuary |  | CUW | Original report or supplemental material |
| Bradley et al. (2020) | USGS Puget Sound Lowland Streams | 2015 | Water | fresh water |  | USGS | NA |
| Conn et al. (2020) | USGS Sand Lance from multiple sites - chlorinated parrafins and alkylphenols | 2010-2014 | Tissue | whole | *Ammodytes personatus* | SGS-AXYS | NA |
| James et al. (2020)^1^ | WDFW 2012-2013 Pilot Study of contaminants in bay mussels from Puget Sound, WA* | 2013 | Tissue | whole | *Mytilus trossulus* | SGS-AXYS | Repository |
| O'Neill et al. (2015)  ^1^ | WDFW 2013 survey of contaminants in juvenile Chinook salmon from Puget Sound, WA.* | 2013 | Tissue | whole | *Oncorhynchus tshawytscha* | SGS-AXYS | Repository |
| Puget Sound Partnership (2023d)^1^ | WDFW 2016 survey of contaminants in juvenile Chinook salmon, WA.* | 2016 | Tissue | whole | *Oncorhynchus tshawytscha* | SGS-AXYS | Repository |
| Puget Sound Partnership (2023c)^1^ | WDFW 2017 Survey of contaminants in English sole from Puget Sound, WA.* | 2017 | Tissue | muscle | *Parophrys vetulus* | SGS-AXYS | Repository |
| Puget Sound Partnership (2023a)^1^ | WDFW 2018 Survey of contaminants in Pacific herring from Puget Sound, WA.* | 2018 | Tissue | whole | *Clupea pallasii* | SGS-AXYS | Repository |
| Puget Sound Partnership (2023b)^1^ | WDFW 2016-2017 Survey of contaminants in subadult resident Chinook salmon from Puget Sound, WA* | 2016-2017 | Tissue | muscle | *Oncorhynchus tshawytscha* | SGS-AXYS | Repository |
| NMFS (2021)^1^ | WDFW 2018 Survey of contaminants in juvenile Chinook from the Green/Duwamish River, Puget Sound, WA* | 2018 | Tissue | whole | *Oncorhynchus tshawytscha* | SGS-AXYS | Repository |
| Langness et al. (2022)  ^1^ | WDFW 2019/2020 Survey of contaminant in transplanted caged bay mussels in Puget Sound, WA.* | 2020 | Tissue | whole | *Mytilus trossulus* | SGS-AXYS | Repository |
| da Silva et al., 2013; and unpublishedRepository^1^ | WDFW 2011-2019 English sole bile survey from Puget Sound, WA* | 2011, 2013, 2015, 2017, 2019 | Tissue | Bile | *Parophrys vetulus* | NOAA - NWFSC^2^ | Repository |
| Lubliner et al. (2010) | WDOE Pharmaceuticals and Personal Care Products in Wastewater Treatment Systems* | 2008 | Water | WWTP effluent |  | MEL, SGS-AXYS | Original report or supplemental material |

Abbreviations: University of Washington Tacoma at the Center for Urban Waters, Tacoma, WA; MEL – Manchester Environmental Laboratory (Washington State Department of Ecology); NOAA-NWFSC – National Oceanic and Atmospheric Administration Northwest Fisheries Science Center laboratories, Seattle, WA; SGS-AXYS – SGS-AXYS Analytical, Burnaby, BC; WDFW - Washington Department of Fish and Wildlife; WDOE - Washington State Department of Ecology; USGS – United States Geological Survey National Water Quality Laboratory, Denver CO.

^1^ The collection of fish and shellfish tissue samples by WDFW employees in the course of their duties is permitted under Washington State Law (RCW [77.12.071](https://urldefense.com/v3/__http:/app.leg.wa.gov/RCW/default.aspx?cite=77.12.071__;!!K-Hz7m0Vt54!gzX4qJT-uXQXRLib-TP5c4hTLICbCcSWjfnM_eLwQb7-3FqOzlrOTmRRM_g7HkX_Fz_KWpRPD8TaXCoqaGAttPqGe0SS$)). WDFW also obtains ESA Section 4(d) and ESA Section 10(a)(1)(A) permits for the collection and incidental take of ESA listed species (e.g., Chinook salmon). For mussel monitoring activities involving deployed caged mussels, WDFW obtains a [Hydraulic Project Approval (HPA)](https://urldefense.com/v3/__http:/wdfw.wa.gov/licensing/hpa/__;!!K-Hz7m0Vt54!gzX4qJT-uXQXRLib-TP5c4hTLICbCcSWjfnM_eLwQb7-3FqOzlrOTmRRM_g7HkX_Fz_KWpRPD8TaXCoqaGAttL9xKyje$), a [Shellfish Transfer Permit](https://urldefense.com/v3/__https:/wdfw.wa.gov/licenses/fishing/shellfish-import-transfer__;!!K-Hz7m0Vt54!gzX4qJT-uXQXRLib-TP5c4hTLICbCcSWjfnM_eLwQb7-3FqOzlrOTmRRM_g7HkX_Fz_KWpRPD8TaXCoqaGAttI92T-4S$), and a Memorandum of Understanding (MOU) with the Washington Department of Natural Resources (DNR) to access [State-Owned Aquatic Lands (SOAL)](https://urldefense.com/v3/__https:/www.dnr.wa.gov/Publications/em_fs11_019_leasing_soal.pdf__;!!K-Hz7m0Vt54!gzX4qJT-uXQXRLib-TP5c4hTLICbCcSWjfnM_eLwQb7-3FqOzlrOTmRRM_g7HkX_Fz_KWpRPD8TaXCoqaGAttABmE-Zs$). WDFW also obtains other permits or permissions (outside those listed above) necessary to conduct mussel monitoring work, including but not limited to site access permits for privately-owned, city, county, port authority, or tribal properties, or state or federal lands.

^2^ Fish bile samples were specifically analyzed for e-EDCs including alkylphenols (NP, 4-nonylphenol triethoxylate, 4-tert-octylphenol (tOP), 4-tert-octylphenol triethoxylate, 4-tert-octylphenol diethoxylate, 4-tert Octylphenol monoethoxylate, and 4-tert-octylphenol triethoxylate), bisphenols (BPA), bisphenol AF (BAF), bisphenol F (BPF), bisphenol S (BPS), and tetrabromobisphenol A), and steroidal estrogens (estrone (E1), estradiol (E2), estriol (E3), and 17α-ethynylestradiol (EE2)) at NOAA’s Northwest Fisheries Science Center. Not all e-EDCs were analyzed by this technique in all sampling years.

**References**

Bradley PM, Journey CA, Button DT, Carlisle DM, Huffman BJ, Qi SL, et al. Multi-region assessment of pharmaceutical exposures and predicted effects in USA wadeable urban-gradient streams. PLOS ONE 2020; 15: e0228214.

Conn KE, Liedtke TL, Takesue RK, Dinicola RS. Legacy and current-use toxic contaminants in Pacific sand lance (Ammodytes personatus) from Puget Sound, Washington, USA. Marine Pollution Bulletin 2020; 158: 111287.

Da Silva, D. A. M., Buzitis, J., Reichert, W. L., West, J. E., O’Neill, S. M., Johnson, L. L., Collier, T. K., & Ylitalo, G. M. (2013). Endocrine disrupting chemicals in fish bile: A rapid method of analysis using English sole (Parophrys vetulus) from Puget Sound, WA, USA. *Chemosphere*, *92*(11), 1550–1556. https://doi.org/10.1016/J.CHEMOSPHERE.2013.04.027

Jack R and Grassley M. Water Quality Assessment and Monitoring Study: Contaminants of Emerging Concern. King County Department of Natural Resources and Parks, Seattle, WA, 2017.

James CA, Lanksbury J, Khangaonkar T, West J. Evaluating exposures of bay mussels (Mytilus trossulus) to contaminants of emerging concern through environmental sampling and hydrodynamic modeling. Science of The Total Environment 2020; 709: 136098.

Langness MM, Nordstrom DL, West JE. Stormwater Action Monitoring 2019/2020 Mussel Monitoring Survey Final Report. Washington Department of Fish and Wildlife, Olympia, WA, 2022.

Lanksbury J. Chemicals of Emerging Concern in Marine and Freshwater Fish in King County. King County Water and Land Resources Division, Seattle, WA, 2022.

Lubliner B, Redding M, Ragsdale D. Pharmaceuticals and Personal Care Products in Municipal Wastewater and Their Removal by Nutrient Treatment Technologies. Publication Number 10-03-004. Washington State Department of Ecology, Olympia, WA, 2010.

Meador JP, Yeh A, Young G, Gallagher EP. Contaminants of emerging concern in a large temperate estuary. Environmental Pollution 2016; 213: 254-267.

NMFS. Lower Duwamish River Natural Resource Damage Assessment: Juvenile Chinook Salmon Growth and Contaminant Exposure Evaluation. Quality Assurance Project Plan (QAPP) and Field Sampling Plan (FSP). National Oceanic and Atmospheric Administration National Marine Fisheries Service (NMFS), Northwest Fisheries Science Center., Seattle, WA, 2021.

O'Neill SM, Carey AJ, Lanksbury JA, Niewolny LA, Ylitalo GM, Johnson LL, et al. Toxic contaminants in juvenile Chinook salmon (Oncorhynchus tshawytscha) migrating through estuary, nearshore and offshore habitats of Puget Sound. . Washington Department of Fish and Wildlife, Olympia, WA, 2015.

Puget Sound Partnership. Toxics in Aquatic Life Indicator - Pacific herring. 2023a. <https://vitalsigns.pugetsoundinfo.wa.gov/VitalSignIndicator/Detail/50>.

Puget Sound Partnership. Toxics in Aquatic Life Indicator - resident Chinook. 2023b.

Puget Sound Partnership. Toxics in Aquatic Life Vital Sign Indicator - English sole. 2023c. <https://vitalsigns.pugetsoundinfo.wa.gov/VitalSignIndicator/Detail/48>.

Puget Sound Partnership. Toxics in Aquatic Life Vital Sign Indicator - juvenile Chinook. 2023d. <https://vitalsigns.pugetsoundinfo.wa.gov/VitalSignIndicator/Detail/49>.

Tian Z, Peter KT, Gipe AD, Zhao H, Hou F, Wark DA, et al. Suspect and Nontarget Screening for Contaminants of Emerging Concern in an Urban Estuary. Environmental Science & Technology 2020; 54: 889-901.
