# Supplementary material for: Estrogenic activity of mixtures in the Salish Sea: The use of high throughput toxicity data with chemical information from fish bile and other matrices
Source: PLoS One. 2025 Jul 9;20(7):epone.0323865. doi: 10.1371/journal.pone.0323865 (PMC12240389; doi:10.1371/journal.pone.0323865)
Supplement: S1 Text — (DOCX) [file pone.0323865.s002.docx]

**S1 Text Data QAQC Review Notes**

Data were obtained from published reports, publications in the peer-reviewed literature, and analytical laboratory reports. In all cases, measured concentrations (not averages or estimates) for individual samples were used and data reporting was accompanied by laboratory quality assurance information, and sample information such as date and location of sample collection, sample matrix, species (as applicable), sample mass or volume, whether a sample was and individual or composite, and number of individuals in a composite (as appropriate). All data were compiled into a single spreadsheet and reviewed for quality assurance prior to use.

Data quality assurance included a review of the laboratory flags, which are notations provided by analytical laboratories to provide the user information necessary for use evaluation. Laboratory flags and the use interpretation are shown below.

| Flags | Description | Included (Y/N) |
| --- | --- | --- |
| D | Dilution data | Y |
| J | Concentration less than LMCL | Y |
| B | Found in sample blank | Y (with blank corrections as indicated below) |
| N | Recovery not within limits | Y (after review) |
| V | Surrogate recovery not within limits | Y (after review) |
| G | Lock max interference | Y |
| NA* | Not Analyzed | N |
| NQ* | Data not quantifiable | N |
| OLR* | Outside linear calibration range | N |
| H | Estimate | Y |
| Max* | Concentration is estimated maximum value | N |
| U* | Not detected | N |
| K* | Peak detected but did not meet quantification criteria, result reported represents the estimated maximum possible concentration | N |

Based on our data review, there were some compounds that were detected in the laboratory blanks. Blanks are essentially clean samples (either pure water from the laboratory pure water system, or vegetable oil that has been certified to be clean by a test lab) that are run through the same processes as the field samples that provide information on systematic laboratory contamination. The presence of a compound in the blanks creates uncertainty as to whether a detection in a field sample is “real” or just a result of lab processing. In order to ensure that detections in field samples are “real,” we did the following: 1) compiled analytical results for all blanks, 2) for those compounds present in three or more blanks, we estimated a distribution of concentrations using mean and standard deviation, 3) we calculated the 95^th^ percentile confidence interval of the concentration in the blanks, and 4) adjusted the analyte reporting limit to be the upper 95^th^ percentile of the distribution. If a field sample concentration was above the upper 95^th^ percentile concentration for the blanks, we deemed that a “real” occurrence. If a field sample concentration was below the upper 95^th^ percentile concentration for the blanks, the concentration estimate was adjusted using half of the adjusted reporting limit.

There were two e-EDCs with blank detections in mussel and fish tissue. Information on the e-EDCs and the adjusted detection limit can be found below.

| Chemical Name | CAS | Adjusted Detection Limit (ng/g) |
| --- | --- | --- |
| 4-Nonylphenol | 104-40-5 | 25.9 |
| 4-n-Octylphenol | 1806-26-4 | 5.8 |
