# Supplementary material for: Estrogenic activity of mixtures in the Salish Sea: The use of high throughput toxicity data with chemical information from fish bile and other matrices
Source: PLoS One. 2025 Jul 9;20(7):epone.0323865. doi: 10.1371/journal.pone.0323865 (PMC12240389; doi:10.1371/journal.pone.0323865)
Supplement: S3 Table — Unless stated otherwise, ER agonists assays were identified by Judson et al. (2015). (DOCX) [file pone.0323865.s004.docx]

|  | | | |  |  |  |  |  |  |
| --- | --- | --- | --- | --- | --- | --- | --- | --- | --- |
| Assay Name | Assay Source | Gene Target | Assay Design | Biological Process | Timepoint (hr) | Organism | Tissue | Cell Format | Cell Line Name |
| NVS_NR_bER | Novascreen | ESR1 | Radioligand binding | Receptor binding | 18 | Bovine | Uterus | Cell-free | NA |
| NVS_NR_hER | Novascreen | ESR1 | Radioligand binding | Receptor binding | 18 | Human | NA | Cell-free | NA |
| NVS_NR_mERa | Novascreen | ESR1 | Radioligand binding | Receptor binding | 18 | Mouse | NA | Cell-free | NA |
| OT_ER_ERaERa_0480 | Odyssey Thera | ESR1 | Protein fragment complementation | Protein stabilization | 8 | Human | Kidney | Cell line | HEK293T |
| OT_ER_ERaERa_1440 | Odyssey Thera | ESR1 | Protein fragment complementation | Protein stabilization | 24 | Human | Kidney | Cell line | HEK293T |
| OT_ER_ERaERb_0480 | Odyssey Thera | ESR1/ESR2 | Protein fragment complementation | Protein stabilization | 8 | Human | Kidney | Cell line | HEK293T |
| OT_ER_ERaERb_1440 | Odyssey Thera | ESR1/ESR2 | Protein fragment complementation | Protein stabilization | 24 | Human | Kidney | Cell line | HEK293T |
| OT_ER_ERbERb_0480 | Odyssey Thera | ESR2 | Protein fragment complementation | Protein stabilization | 8 | Human | Kidney | Cell line | HEK293T |
| OT_ER_ERbERb_1440 | Odyssey Thera | ESR2 | Protein fragment complementation | Protein stabilization | 24 | Human | Kidney | Cell line | HEK293T |
| OT_ERa_EREGFP_0480 | Odyssey Thera | ESR1 | Fluorescent protein induction | Regulation of gene expression | 8 | Human | Cervix | Cell line | HeLa |
| OT_ERa_EREGFP_0120 | Odyssey Thera | ESR1 | Fluorescent protein induction | Regulation of gene expression | 2 | Human | Cervix | Cell line | HeLa |
| ATG_ERE_CIS_up | Attagene | ESR1 | mRNA induction | Regulation of transcription factor activity | 24 | Human | Liver | Cell line | HepG2 |
| ATG_ERa_TRANS_up | Attagene | ESR1 | mRNA induction | Regulation of transcription factor activity | 24 | Human | Liver | Cell line | HepG2 |
| Tox21_ERa_LUC_VM7_Agonist^1^ | Tox21 | ESR1 | Lutiferase induction | Regulation of transcription factor activity | 22 | Human | Ovary | Cell line | VM7 |
| Tox21_ERa_LUC_VM7_ICI182780^2^ | Tox21 | ESR1 | Lutiferase induction | Regulation of transcription factor activity | 22 | Human | Ovary | Cell line | VM7 |
| Tox21_ERa_BLA_Agonist_ratio | Tox21 | ESR1 | Beta lactamase induction | Regulation of transcription factor activity | 24 | Human | Kidney | Cell line | HEK293T |
| Tox21_ERb_BLA_Agonist_ratio^3^ | Tox21 | ESR2 | Beta lactamase induction | Regulation of transcription factor activity | 24 | Human | Kidney | Cell line | HEK293T |
| ACEA_ER_80hr^4^ | ACEA Biosciences | ESR1 | Real-time cell-growth kinetics | Cell proliferation | 80 | Human | Breast | Cell line | T47D |

S3 Table. Summary of ToxCast assays related to the estrogen receptor agonist signaling pathway. Unless stated otherwise, ER agonists assays were identified by Judson et al. (2015). ESR1 and ESR2 are the estrogen receptor 1 and 2 gene targets.

^1^ Tox21_ERa_LUC_VM7_Agonist was previously named Tox21_ERa_LUC_BG1_Agonist as it was previously annotated as being run in BG1 cells, but was recently shown to be of MCF7 origin (Judson et al., 2017)

^2^ Secondary assay developed for Tox21_ERa_LUC_VM7_Agonist. Identified as an ER agonist assay in Maloney et al. (2023)

^3^ Assay targeting the ESR2 receptor was new in 2020. Identified as an ER agonist assay in Maloney et al. (2023)

^4^Assay renamed from ACEA_T47D_80hr

**References**

Judson RS, Magpantay FM, Chickarmane V, Haskell C, Tania N, Taylor J, et al. Integrated Model of Chemical Perturbations of a Biological Pathway Using 18 In Vitro High-Throughput Screening Assays for the Estrogen Receptor. Toxicol Sci [Internet]. 2015 [cited 2023 Feb 23];148(1):137–54.

Maloney EM, Villeneuve DL, Jensen KM, Blackwell BR, Kahl MD, Poole ST, et al. Evaluation of Complex Mixture Toxicity in the Milwaukee Estuary (WI, USA) using Whole Mixture and Component‐Based Evaluation Methods. Environ Toxicol Chem [Internet]. 2023 Jan 30 [cited 2023 Mar 7].
