# Supplementary material for: Estrogenic activity of mixtures in the Salish Sea: The use of high throughput toxicity data with chemical information from fish bile and other matrices
Source: PLoS One. 2025 Jul 9;20(7):epone.0323865. doi: 10.1371/journal.pone.0323865 (PMC12240389; doi:10.1371/journal.pone.0323865)
Supplement: S2 Text — (DOCX) [file pone.0323865.s005.docx]

**S2 Text.  ER Agonist Manual Evaluation**

Twenty chemicals without available AUC scores were manually evaluated to predict interaction with the estrogen receptor (ER), with a positive call indicating likely interaction and a negative call indicating unlikely interaction. The following considerations were taken into account to make a putative call:

| Description | Putative Call |
| --- | --- |
| Collaborative Estrogen Receptor Activity Prediction Project (CERAPP) prediction is positive | Positive |
| Only Attagene (ATG) ER assays were active, no positive CERAPP prediction | Negative |
| There is activity in assays outside of the ATG platform, but the maximum activity (Top value or T-value) for chemical-assay response is <50, and/or response is associated with excluded lab flags* | Negative |
| Activity in assays outside of the ATG platform, the associated T-value is >50, and the response is not associated with excluded lab flags* | Positive |

*Excluded lab flags:

| Flag | Description |
| --- | --- |
| Borderline Active | Assay is active with borderline activity |
| Only highest conc above baseline, active | Single point hit with activity only at the highest concentration tested |
| Gain AC50 < lowest conc & loss AC50 < mean conc | Gain-loss model won, though the gain concentration at half-maximal activity (AC50) is less than the minimum tested concentration, and the loss AC50 is less than the mean tested concentration |
| AC50 less than lowest concentration tested | Activity concentration at half-maximal activity is less than the lowest concentration tested |
