# Supplementary figures and images for: Estrogenic activity of mixtures in the Salish Sea: The use of high throughput toxicity data with chemical information from fish bile and other matrices
Source: PLoS One. 2025 Jul 9;20(7):epone.0323865. doi: 10.1371/journal.pone.0323865 (PMC12240389; doi:10.1371/journal.pone.0323865)

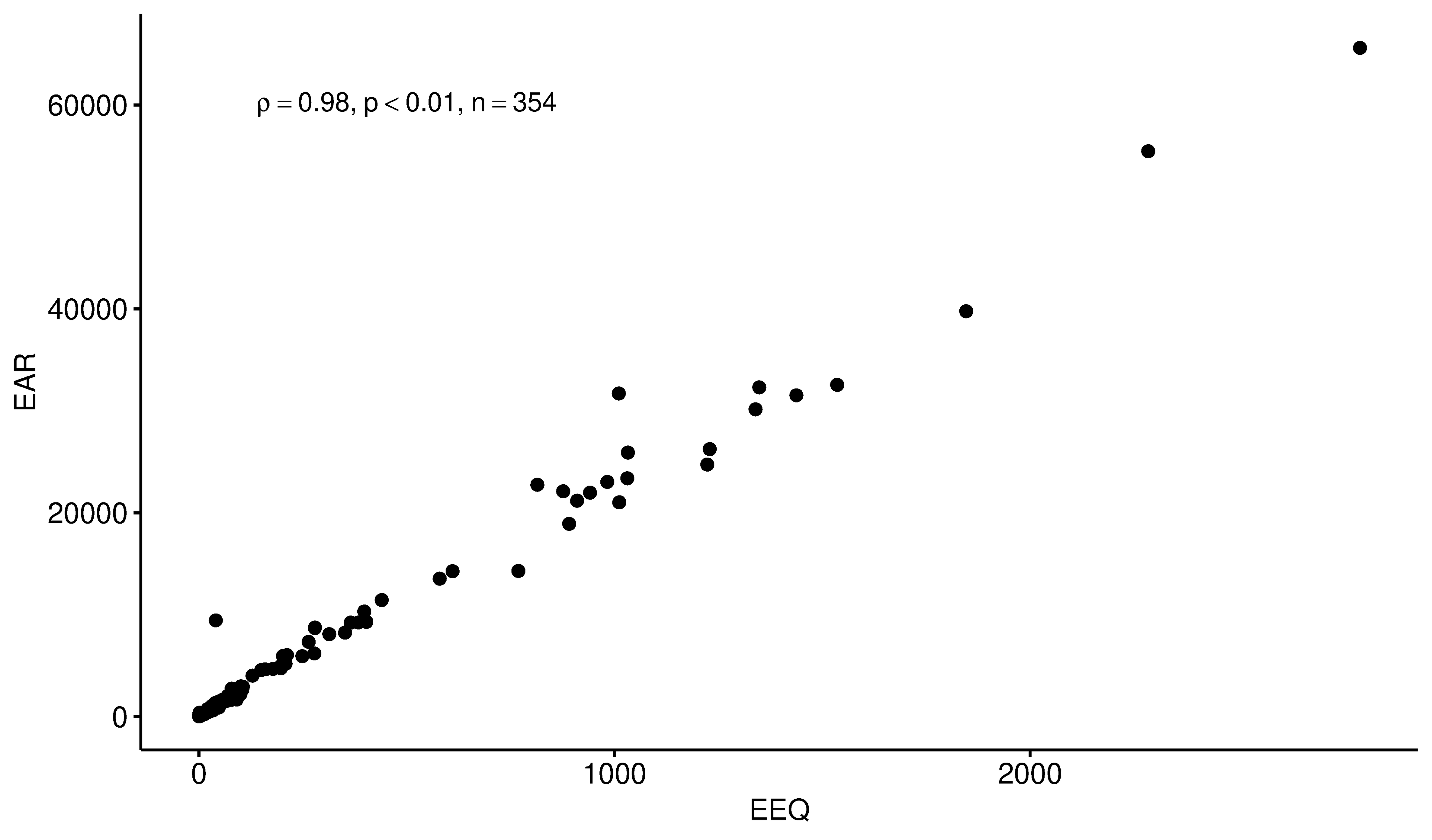

Supplement: S1 Fig — (TIF) [file pone.0323865.s009.tif]

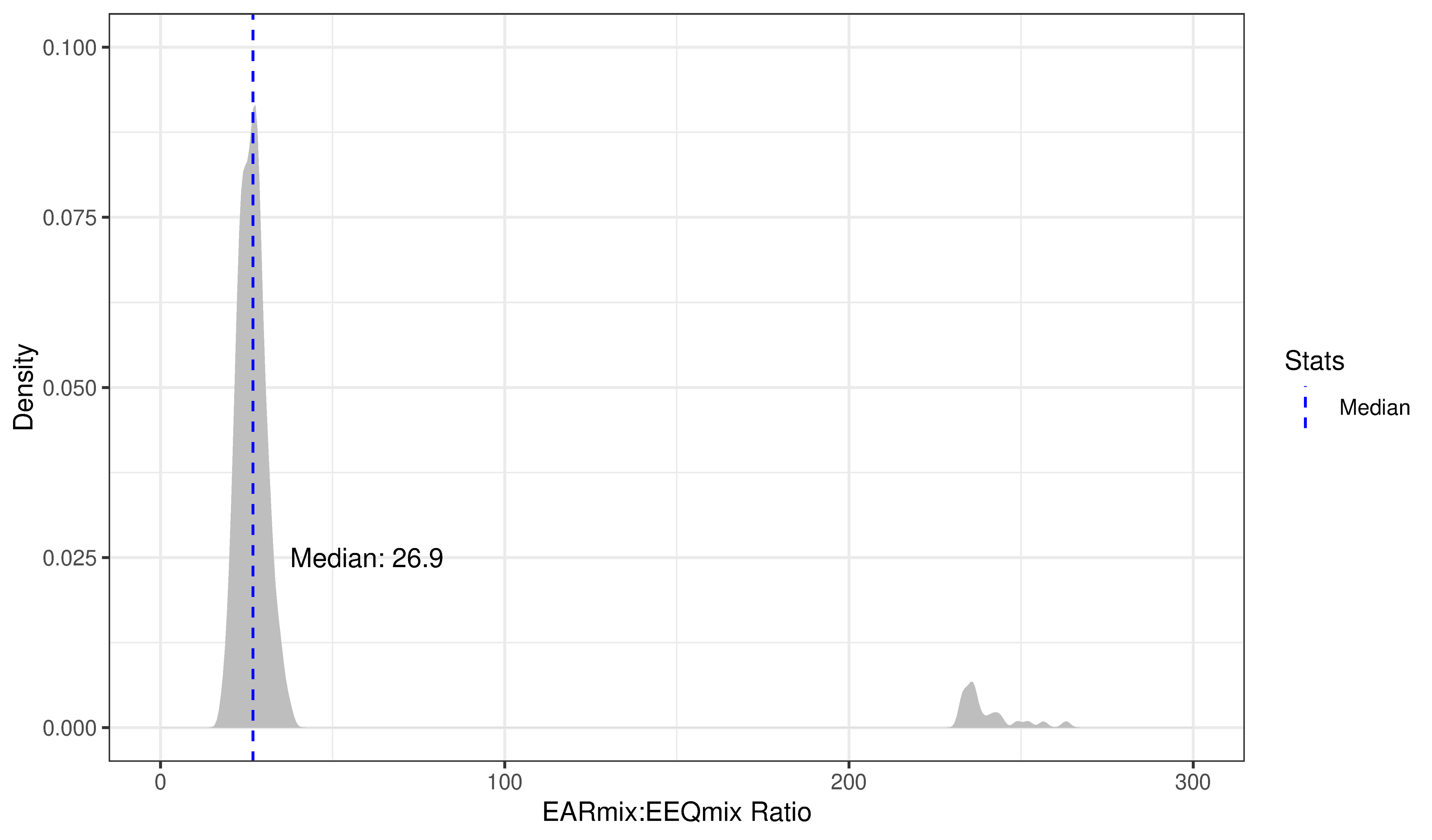

Supplement: S2 Fig — (TIF) [file pone.0323865.s010.tif]

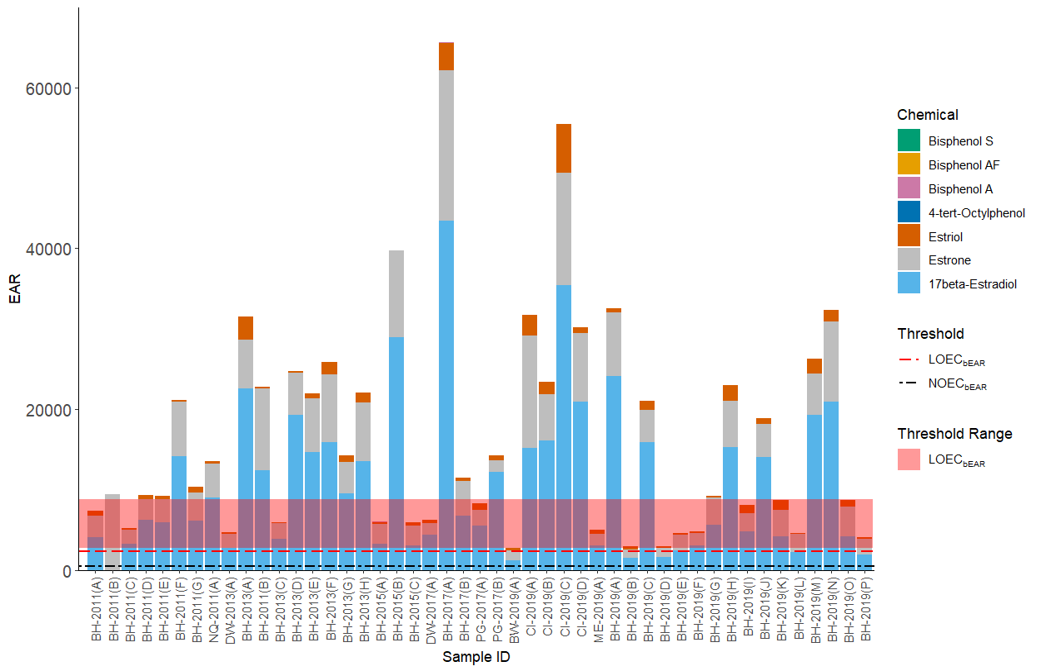

Supplement: S3 Fig — Stacked bar plot with exposure-activity ratios (EARs) for each bile sample with EARs exceeding the LOECbEAR, represented by the red dashed line. The sample labels include the site ID abbreviation (as described in Fig 1), collection year, and a letter to distinguish samples. The EARACC5 for each chemical and the overall EARmix for the sample are shown. The dotted and dashed line represents the lowest NOECbEAR, and the dashed line represents the lowest LOECbEAR calculated using the lowest BCFbw. The shaded regions represent the range of calculated possible threshold values based on range of BCFbw values. (TIF) [file pone.0323865.s012.tif]

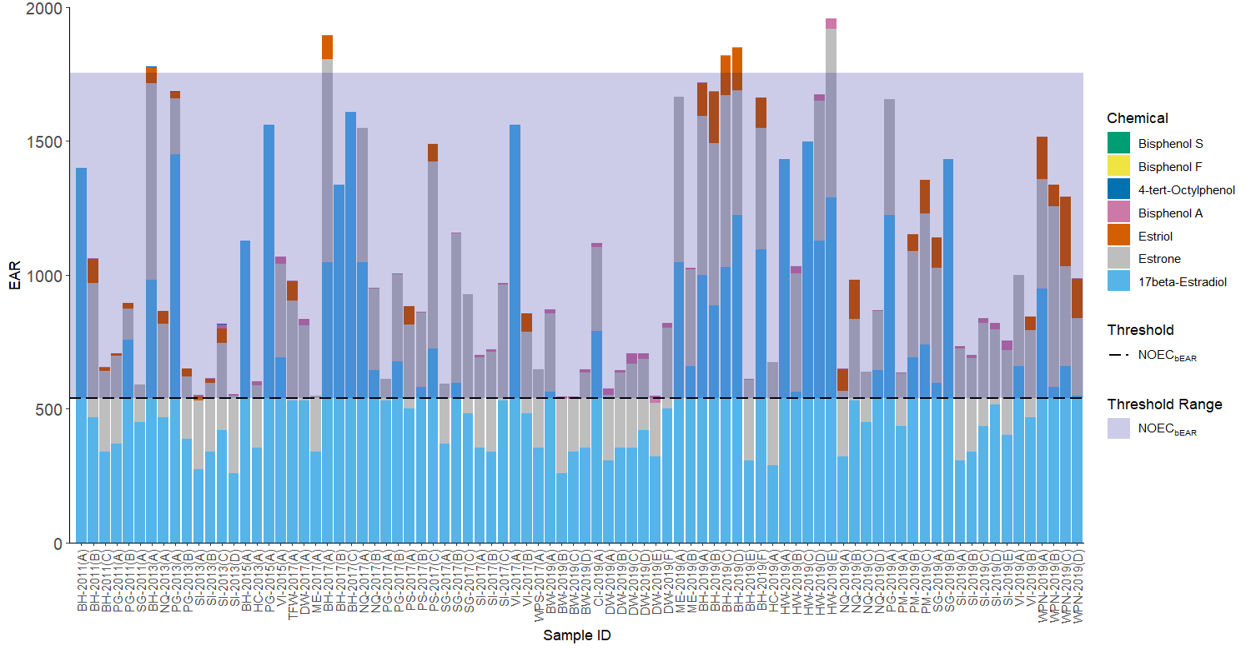

Supplement: S4 Fig — Stacked bar plot with exposure-activity ratios (EARs) for each bile sample with EARs between the LOECbEAR and the NOECbEAR, represented by the black dashed line. The sample labels include the site ID abbreviation (as described in Fig 1), collection year, and a letter to distinguish samples. The EARACC5 for each chemical and the overall EARmix for the sample are shown. The the shaded region represents the range of calculated possible NOEC threshold values based on range of BCFbw values. (TIF) [file pone.0323865.s013.tif]
